# Role of anoikis-related gene RAC3 in prognosis, immune microenvironment, and contribution to malignant behavior *in vitro* and *in vivo* of bladder urothelial carcinoma

**DOI:** 10.3389/fphar.2024.1503623

**Published:** 2024-11-26

**Authors:** Yusong Zhou, Shiwei Huang, Bing Yang, Jing Tan, Zhun Zhang, Wei Liu

**Affiliations:** ^1^ Department of Pharmacy, The Third Xiangya Hospital, Central South University, Changsha, China; ^2^ Department of Urology, The Third Xiangya Hospital, Central South University, Changsha, China; ^3^ Department of Pharmacy, Zunyi Medical University, Zunyi, China; ^4^ Department of Breast and Thyroid Surgery, The Third Xiangya Hospital, Central South University, Changsha, China

**Keywords:** anoikis, Bladder urothelial carcinoma, RAC3, prognosis, immune microenvironment, proliferation, migration

## Abstract

**Background:**

Anoikis disrupts the normal apoptotic process in cells, leading to abnormal proliferation and migration, thereby promoting tumor formation and development. However, the role of anoikis in bladder urothelial carcinoma (BLCA) still requires further exploration.

**Methods:**

Anoikis-related genes (ARGs) were retrieved from the GeneCards and Harmonizome databases to distinguish various subtypes of BLCA and develop a predictive model for BLCA. The immune microenvironment and enrichment pathways between various subtypes were also analyzed using consensus clustering. Potential medications were screened by utilizing drug sensitivity analysis. *In vitro* and vivo, the character of the independent prognostic gene in BLCA was confirmed through cell studies and mouse xenograft models.

**Results:**

One hundred thirty differentially expressed genes (DEGs) were identified, and nine of them were chosen to construct predictive models that can accurately forecast the prognosis of BLCA patients. K = 2 was correctly identified as the optimal clustering type for BLCA, showing prominent differences in survival rates between the two subgroups. The immune-related functional studies manifested that the two subtypes’ immune cell expressions differed. It was verified that RAC3 is an independent prognostic gene for BLCA. RAC3 shows high expression levels in BLCA, as indicated by its consistent mRNA and protein levels across different gene expressions. The functional verification results of RAC3 in BLCA showed that silencing RAC3 can significantly inhibit BLCA cell proliferation, colony formation, and migration. RAC3 knockdown inhibited the growth and migration of BLCA *in vivo*. SB505124 exhibited a significant inhibitory effect on the proliferation of BLCA cells.

**Conclusion:**

Based on the predictive model developed in this study, BLCA patients’ prognoses can be accurately predicted. SB505124 could become an important drug in the treatment of BLCA patients. RAC3 is essential in prognosis, immune microenvironment, and malignant behavior of BLCA *in vitro* and *in vivo*. It will also offer the potential for personalized treatment for BLCA patients and generate new research avenues for clinical investigators.

## 1 Introduction

Bladder urothelial carcinoma (BLCA) occurs primarily in the urinary tract’s intestinal mucosa (Himura et al., 2024). BLCA is insidious in its onset, highly invasive (Zhang et al., 2022), and prone to metastasize even after surgery because of its high recurrence rate (Liu et al., 2024). Approximately 75% of individuals receive a diagnosis of non-muscle-invasive bladder cancer (NMIBC), with the remaining patients being diagnosed with muscle-invasive bladder cancer (MIBC) (Cumberbatch et al., 2018). Despite the favorable prognosis for patients with NMIBC, the majority of patients experience recurrence, and a proportion of them progress to MIBC (Nunes et al., 2024). Many patients are diagnosed at advanced stages due to the absence of precise diagnosis and treatment options. For patients with advanced bladder cancer, the risk of metastasis is observably increased, and the prognosis is poor (Ashrafizadeh et al., 2020). Therefore, early diagnosis and tumor evaluation are crucial.

Anoikis’ occurrence process is mainly the separation of epithelial cells and extracellular matrix (ECM), which is a kind of programmed cell death. In tumors, the ECM is often destroyed (Cai and Zhou, 2022). Anoikis is a vital process that halts cell growth and adhesion to the incorrect matrix, thus preventing cells from being transplanted into a distant organ (Paoli et al., 2013). The anoikis pathway includes the intrinsic pathway mediated by mitochondrial damage and the extrinsic pathway mediated by activation of cell surface death receptors. These pathways interact and together activate effectors, causing the anorexia-inducing cascade (Fofaria and Srivastava, 2014), which has been demonstrated to be a critical mechanism in the invasion and spread of cancer and was first examined for its existence in epithelial and endothelial cells (Kakavandi et al., 2018). The development of resistance to anoikis can enable isolated cells to evade the cell death signaling pathway and survive in unfavorable conditions (Adeshakin et al., 2021). Some studies have shown that anoikis plays a crucial role in tumor metastasis and cancer progression, such as in lung (Di Micco et al., 2021), esophageal (Jin et al., 2018), and gastric cancers (Chen et al., 2018), among others. One study identified seven lncRNAs connected to the risk of BLCA. The study also utilized modeling to identify the signaling pathways in which these lncRNAs may be involved and assessed their accuracy in prognostic prediction (Ye et al., 2020). However, there is a lack of sufficient studies on ARGs in BLCA.

Therefore, in our study, we examined the relationship between the ARGs’ expression in BLCA and their potential prognostic and immune correlations. We developed a risk-prognostic model of BLCA by screening for ARGs with significant predictive value. Our study then identifies independent prognostic genes and validates them through *in vitro* cell and animal experiments. We aimed to illuminate the possible impact of ARGs in BLCA. This study has a significant guiding value for the clinical treatment and prognosis of BLCA.

## 2 Materials and methods

### 2.1 Data acquisition and preliminary processing

The RNA sequences of BLCA patients (including 412 tumor samples and 19 normal samples) and related clinical information data used for analysis were downloaded from The Cancer Genome Atlas (TCGA) database (https://portal.gdc.cancer.gov/), seeing the Supplementary Table S1 from the relevant information. The initial count data was transformed into transcripts per million (TPM) and subsequently converted to log2 for further analysis. The GSE32548 dataset was sourced from the Gene Expression Omnibus (GEO, https://www.ncbi.nlm.nih.gov/geo/) database and contains clinical pathological information for 131 tumor patients. This dataset underwent both quantile normalization and log2 transformation before being analyzed. Somatic mutation counts and copy number variation (CNV) also were downloaded from the TCGA database.

### 2.2 Screening of DEGs and comparison of mutation frequencies

Screening for ARGs was performed using the GeneCards database (https://www.genecards.org/) with a correlation score greater than 0.4 as the screening condition, in conjunction with the Harmonizome database (https://maayanlab.cloud/Harmonizome/) (Supplementary Table S2). First, utilize the “limma” and “pheatmap” packages to conduct a differential analysis and identify distinctions between tumor and normal tissue in BLCA patients. Subsequently, prognostic analysis was performed using univariate Cox regression analysis to screen out DEGs with prognostic value. These genes were visualized using the “igraph,” and “RColorBrewer” packages to show the connections between DEGs. The cell mutation rate, genetic site, and the CNVs of these genes are analyzed using the “Rcirco” R package, which generates a series of visualizations.

### 2.3 Clustering and immune-related functional analysis

Unsupervised subgroups and clusters of the TCGA-BLCA dataset were identified using the R package “ConsensusClusterPlus” (Wilkerson and Hayes, 2010) based on overlapping ARGs. Clustering was verified by principal component analysis (PCA) using the R packages “broom,” “Rtsne” and “umap” respectively. Kaplan-Meier survival curves for different subgroups and clusters were analyzed and plotted using the R packages “survival” and “survminer.” Gene expression of different subgroups and clusters was visualized using the R package “Heatmap.” Following this, R package “clusterProfiler” was performed for functional or pathway enrichment analysis of DEGs (Bian et al., 2022), the Kyoto Encyclopedia of Genes and Genomes (KEGG) pathway and Reactome pathway were analyzed using the R package “GSVA” (Hänzelmann et al., 2013) and “msigdbr” to explore the differences in biological processes between different subgroups. Use the ssGSEA algorithm to study the immune cell infiltration relationship between different subgroups. The infiltration of immune cells in different subgroups was visualized using the R package “ggplot2.”

### 2.4 Building a risk-scoring model

The basic genetic factor of LASSO regression is used to forecast the survival and outlook of BLCA. The formula used for calculating the risk score is as follows: Risk score = $\sum_{i=1}^{n}\exp-genei*coef-genei$ . Using the TCGA-BLCA dataset, patients were classified into high- or low-risk categories based on the median risk score. Patients were divided into a training group and a test group at a ratio of 1:1 to verify the survival prediction of BLCA patients. The receiver operating characteristic (ROC) curves can forecast the survival rates of BLCA patients at 1, 3, and 5 years. The predictive effect of the risk-scoring model is evaluated by analyzing the survival model. The nomogram displays the outcomes of the Cox regression, formulating a scoring standard based on the magnitude of the regression coefficient for all predictive indicators. Each level of the prediction index corresponds to a score, and adjustments can be made on a specific scale to accommodate the interrelationships between each variable in the prediction model. Subsequently, the probability of a clinical outcome for each patient can be calculated based on the score. The display format draws a line with scales on the nomogram. Sankey plots were applied using the R packages “highcharter”, “ggplot2” and “ggalluvial” to display cluster distributions of risk groups and survival outcomes.

### 2.5 Immune microenvironment analysis and screening potential therapeutic drugs

Risk scores were compared to immune cell infiltration by utilizing the CIBERSORT algorithm to assess the levels of immune cells (ImmuneScores) and stromal cells (StromalScores). BLCA’s stromal, immune, and ESTIMATE scores were calculated with the R software package “estimate”. By using the “CIBERSORT R script v1.03”, it is possible to decide the percentage of 22 different types of immune cells in a given sample. Data on drug response and drug-targeted pathways were gathered from the Genomics of Drug Sensitivity in Cancer (GDSC, https://www.cancerrxgene.org/(accessed 22 August 2023)) to examine variations in sensitivity to chemotherapeutic drugs among groups with high and low-risk scores. Next, data on drugs and drug targeting pathways were gathered utilizing the R packages “ggalluvial,” “ggplot2,” “dplyr,” and “ggpubr.” The R packages are created to perform correlation analyses on drug data and risk scores, investigate the connection between risk scores and drug responsiveness, and link them to the signaling pathways targeted by these drugs.

### 2.6 Identifying genes that can predict outcomes independently

Survival differences between the two groups were compared utilizing the Kaplan-Meier (K-M) method to conduct survival analysis. The log-rank test was utilized to generate *p*-values, hazard ratio (HR), and 95% confidence interval (CI) for high and low expression of prognostic genes. The “forestplot” package was utilized to create a forest plot that visualizes the *p*-values, HR, and 95% CIs for each variable in univariate and multivariate Cox regression analyses to identify the separate predictive genes of interest. The expression of separate predictive genes in BLCA and normal samples was also shown.

### 2.7 *In vivo* tumor models

Female BALB/c nude mice, aged between five and 6 weeks, were acquired from SJA Laboratory Animal Co., Ltd. and kept in conditions free of specific pathogens. The housing conditions included a controlled temperature range of 22°C–26°C, humidity at 55% ± 5%, and a 12-h light/dark cycle. Each cage housed 5 mice. Every animal study followed the guidelines outlined in the legislation of the People’s Republic of China concerning the use and treatment of animals in laboratories. Random assignment of mice to experimental groups was conducted. To establish xenograft models, mice were subcutaneously injected with a suspension of T24-shCtrl or T24-ShRAC3-2 cells. The formula used to calculate tumor volumes was 1/2 multiplied by the long diameter multiplied by the square of the short diameter. Tumor volumes and mouse weights were measured every 2 days once the tumors reached a size of 70–100 cm^3^.

### 2.8 Sources of tissues

From June to December 2023, 12 pairs of clinical tissue specimens from BLCA and nearby normal tissues were gathered at the Third Xiangya Hospital of Central South University, which were mainly utilized for functional validation to explore the expression level of RAC3.

### 2.9 Immunohistochemical staining

Immunoblot was performed as previously described (Li et al., 2022), and the expression levels of important genes in different tissues were found. The tissues were encased in paraffin, cut into 6 μm sections, and preserved in 4% paraformaldehyde. After deparaffinizing, the sections were rehydrated in preparation for immunohistochemistry staining. A Tris-EDTA buffer solution (10 mM Tris-HCl and 1 mM EDTA) was used on the rehydrated sections to deactivate the natural peroxidase and eliminate the antigen. For five minutes, the parts were heated to boil in an autoclave. After three washes, the samples were then exposed to BSA for half an hour and left overnight at 4°C with the primary antibody (Supplementary Table S3). The sections were incubated with a second antibody for one hour at room temperature the next day. After staining the nucleus with hematoxylin, the sections were air-dried and mounted using neutral resins.

### 2.10 qPCR

Total RNA was isolated using the RNeasy Mini Kit (QIAGEN, Beijing, China). Using a high-capacity cDNA reverse transcription kit (Thermo, Shanghai, China), complementary DNA (cDNA) was created. PCR was performed using TaqMan Human RAC3 and GAPDH Probes, as well as TaqMan Gene Expression Master Mix, both from Sangon Biotech in Shanghai, China. The primers used in this study include RAC3 (forward 5′-TCC​CCA​CCG​TTT​TTG​ACA​ACT-3′, reverse 5′-GCA​CGA​ACA​TTC​TCG​AAG​GAG-3′) and GAPDH (forward 5′-CAG​GAG​GCA​TTG​CTG​AT-3′, reverse 5′-GAA​GGC​TGG​GCT​CAT​TT-3′).

### 2.11 Western blot

After transfecting cells with RAC3 or control plasmids, proteins were extracted 48 h later. Protein extracts were then separated utilizing SDS-PAGE, transferred to the PVDF membrane, and detected using RAC3 primary antibodies (Supplementary Table S3). Antigen-antibody reactions were observed using the ChemiDoc system with peroxide-conjugated secondary antibodies. Band intensity was quantified using Image J.

### 2.12 MTT

Following a 24-h transfection with either the RAC3 or control plasmid (Supplementary Table S4), cells were seeded into 96-well plates (6 × 10^3^/well). Add 50 μM of 2 mg/mL MTT to each well, then let it sit for 4 h in the incubator. Once the medium has been removed, add 150 μL of DMSO and shake for ten minutes. The wavelength at which absorbance (A) is measured is 490 nm.

### 2.13 Cologenic assay

After giving cells, a 24-h transfection with RAC3 or a control plasmid, seed the cells into 24-well plates (1 × 10^3^/well). Fix the cells in the wells with a 10% formaldehyde solution after 6–8 days 0.1% crystal violet was applied at the end to stain the cells. The wavelength at which absorbance is measured is 550 nm.

### 2.14 Scratch assay

Seed the cells into 12-well plates (3 × 10^5^/well) after 24 h of RAC3 or control plasmid transfection. After a day, use a 200-μL tip to scrape the cells and make a damaged area. Rinse twice with saline that has been buffered with phosphate. Cells are grown for a specified time in a medium without serum. Under a microscope, digital pictures were taken at 0 and 24 h.

### 2.15 Transwell assay

After transfecting the cells with either the RAC3 or control plasmid, they were left to incubate for 24 h, 4 × 10^4^ cells were placed into the upper chamber with 400 μL of serum-free medium, along with 600 μL of the chemotaxis-enhancing medium. The cells were treated with 0.1% crystal violet and fixed with 4% paraformaldehyde after 24 h. Remove the non-invasive cells from the membrane’s upper side using absorbent cotton. Then, take pictures under a microscope.

### 2.16 CD8^+^ T cell cytotoxic assay

CD8^+^ T cells were obtained according to the methods reported in the literature (Shen et al., 2022). Tumor cells were subjected to transfection with RAC3 or plasmid control and subsequently incubated for 24 h. Following this incubation period, they were plated in a 96-well plate with a density of 10,000 cells per well. After 12 h, CD8^+^ T cells were added to the 96-well plate. After co-incubation for 48 h, the culture medium was removed, and the tumor cells were washed twice with PBS to remove the T cells. Lastly, the killing effect of CD8^+^ T cells on tumor cells was detected by MTT assay.

### 2.17 Statistical analysis

Annotation and curation of transcriptome data, and clinical and gene expression data were performed using PERL (v5.30.0). Other statistical analyses were performed using R software (version 4.3.0; version 4.3.1). The Wilcoxon rank-sum test was used to test the difference between two groups of continuous variables. A *p*-value <0.05 was considered statistically significant.

## 3 Result

### 3.1 Current research process

The flow of this study is shown in Supplementary Figure S1. Our BLCA transcriptome samples mainly come from the TCGA-BLCA data set, which contains 412 tumor samples and 19 normal samples. In addition, 131 tumor samples from GEO-GSE32548 were also included. ARGs were obtained from GeneCards and Harmonisome platforms. First, a differential analysis was performed to identify 130 differentially expressed genes, and univariate Cox regression was performed on these genes to identify 38 genes related to the prognosis of BLCA. These genes were then subjected to consensus clustering classification survival analysis and KEGG enrichment pathway validation. Using LASSO regression analysis and proportional hazards model analysis, we finally selected 9 genes for prognostic model construction and evaluated the prognosis and immune microenvironment of BLCA. Finally, we performed survival analysis and univariate and multivariate Cox analysis on these 9 genes to screen independent prognostic genes with important value for *in vivo* and *in vitro* validation. It was finally determined that RAC3 is an important independent prognostic gene involved in the proliferation, migration, and invasion of BLCA.

### 3.2 The expression of ARGs in BLCA

**FIGURE 1:**
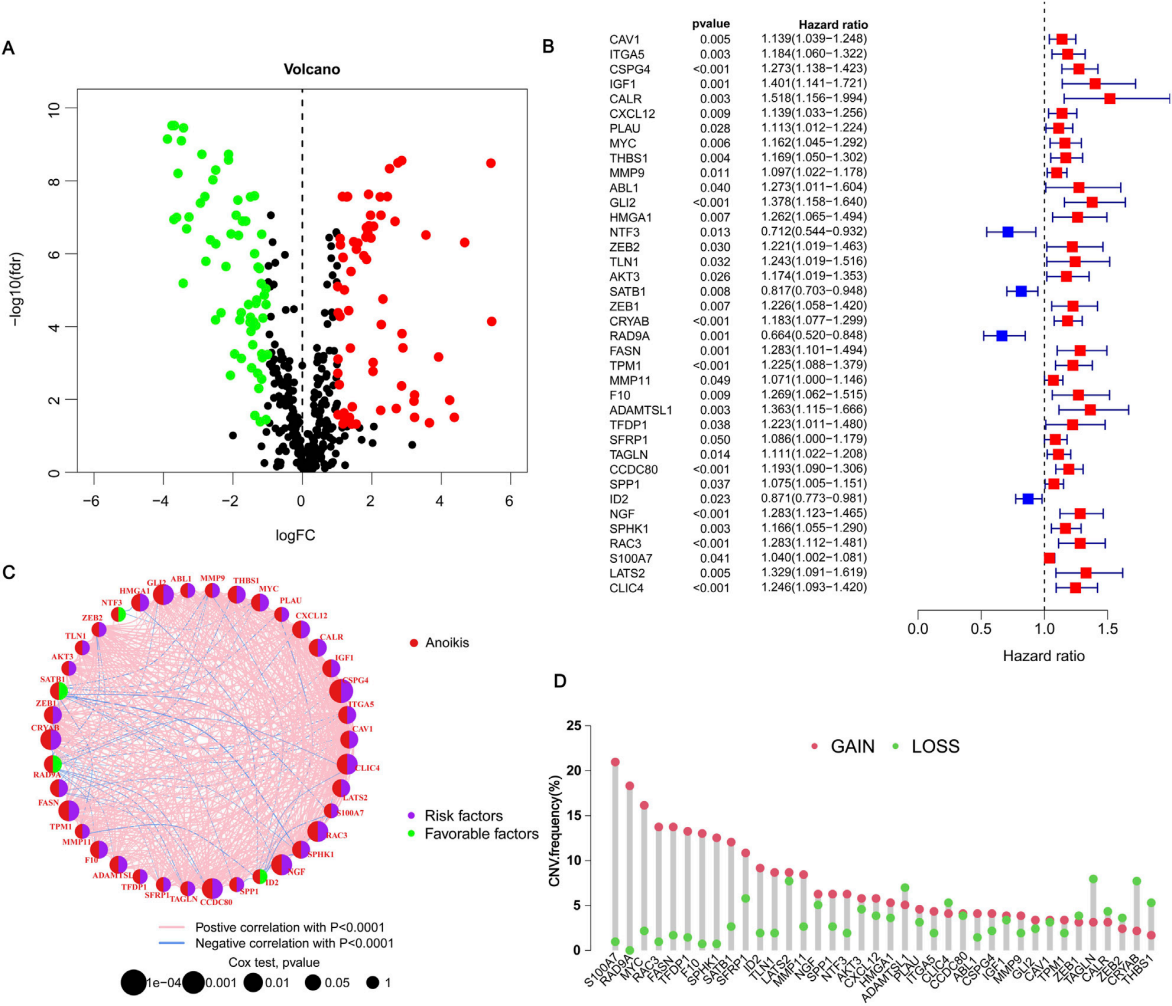
Expression of anoikis-related genes in BLCA. **(A)** Volcano plot showing threshold setting |lgFC| ≥ 1 and *p*-value <0.05. Green represents low-risk genes, and red represents high-risk genes. **(B)** Showing 38 prognosis-related genes by univariate Cox regression analysis. Blue represents HR < 1, indicating a low-risk gene; red represents HR > 1, indicating a high-risk gene. *p* < 0.05 indicates statistical significance. **(C)** Network diagram showing the correlation between 38 genes. Purple indicates that the gene is a risk factor for BLCA, and green indicates that the gene is a favorable factor for BLCA. Pink and blue lines represent positive and negative correlations between genes, respectively. **(D)** Copy number variations (CNVs) frequency of 38 genes in TCGA-BLCA. Red circles represent copy number amplification, and green circles represent copy number deletions.

To verify the differences in ARGs in BLCA, through the “limma” package, we obtained a total of 130 genes expressed in the differences in BLCA and adjacent normal tissues. These genes are called differentially expressed genes (DEGs). The heatmap shows the top 100 critical DEGs that are up or down (Supplementary Figure S2). Of these, including 50 upregulated genes and 50 downregulated genes (Figure 1A). A univariate Cox analysis of the differences mentioned above is performed, and 38 genes related to the prognosis of BLCA are determined (Figure 1B). Among the 38 ARGs, NTF3, SATB1, RAD9A, ID2, etc. al., are labeled as green, HR < 1 means protective factors benefit prognosis. The remaining genes, such as RAC3 are marked purple with HR > 1, indicating poor prognosis (Figure 1C). The differential expression of these genes is very important for tumor classification and diagnosis. Except for ADANMTSL1, CLI4, ZEB1, TAGLN, CALR, ZEB2, CRYAB, and THBS1, the number of copies of other genes increased more than the frequency of the loss of copies (Figure 1D). The positions of these differential genes on 23 pairs of chromosomes were indicated (Supplementary Figure S3). Except for 5, 14, 16, 18, 21, and two sex chromosomes, which did not show mutant genes, the other chromosomes showed mutant genes. The CNV of different genes may provide an important basis for clinical diagnosis, prognosis assessment, and implementation of targeted therapy in different patients.

### 3.3 Consensus clustering and immune microenvironment landscape analysis

**FIGURE 2:**
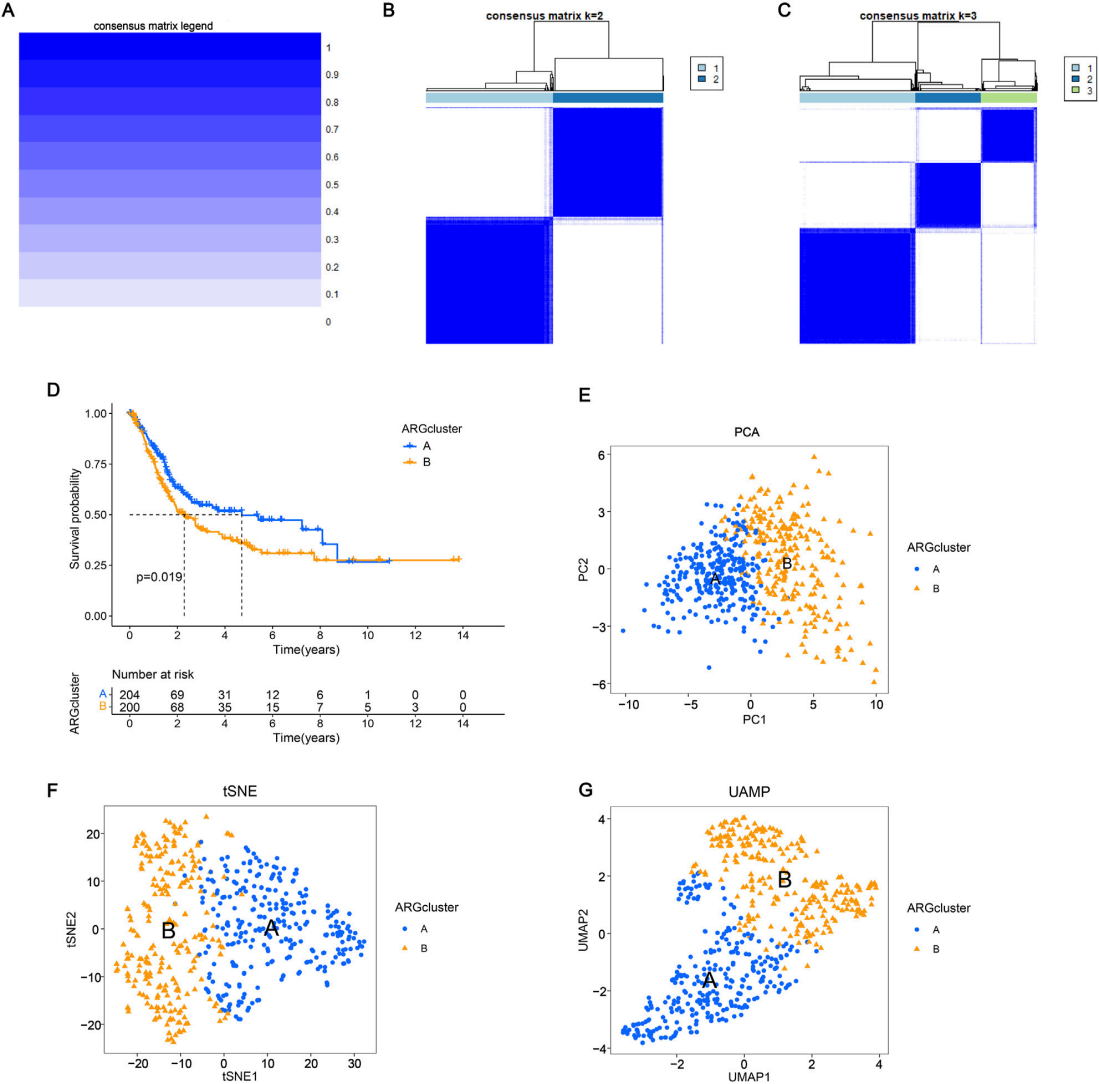
Consensus clustering and immune microenvironment landscape analysis **(A–C)** Consensus matrix depicting the clustering results when k (cluster number) is set to 1–3. The expression k = 2 indicates that the two clusters are optimally classified. **(D)** The K-M curve shows the change in the survival probability of two subgroups over time. **(E–G)** Principal component analysis, T-distributed stochastic neighbor embedding (t-SNE), and UAMP analysis showed significant differences in the distribution of patients in groups A and B.

**FIGURE 3:**
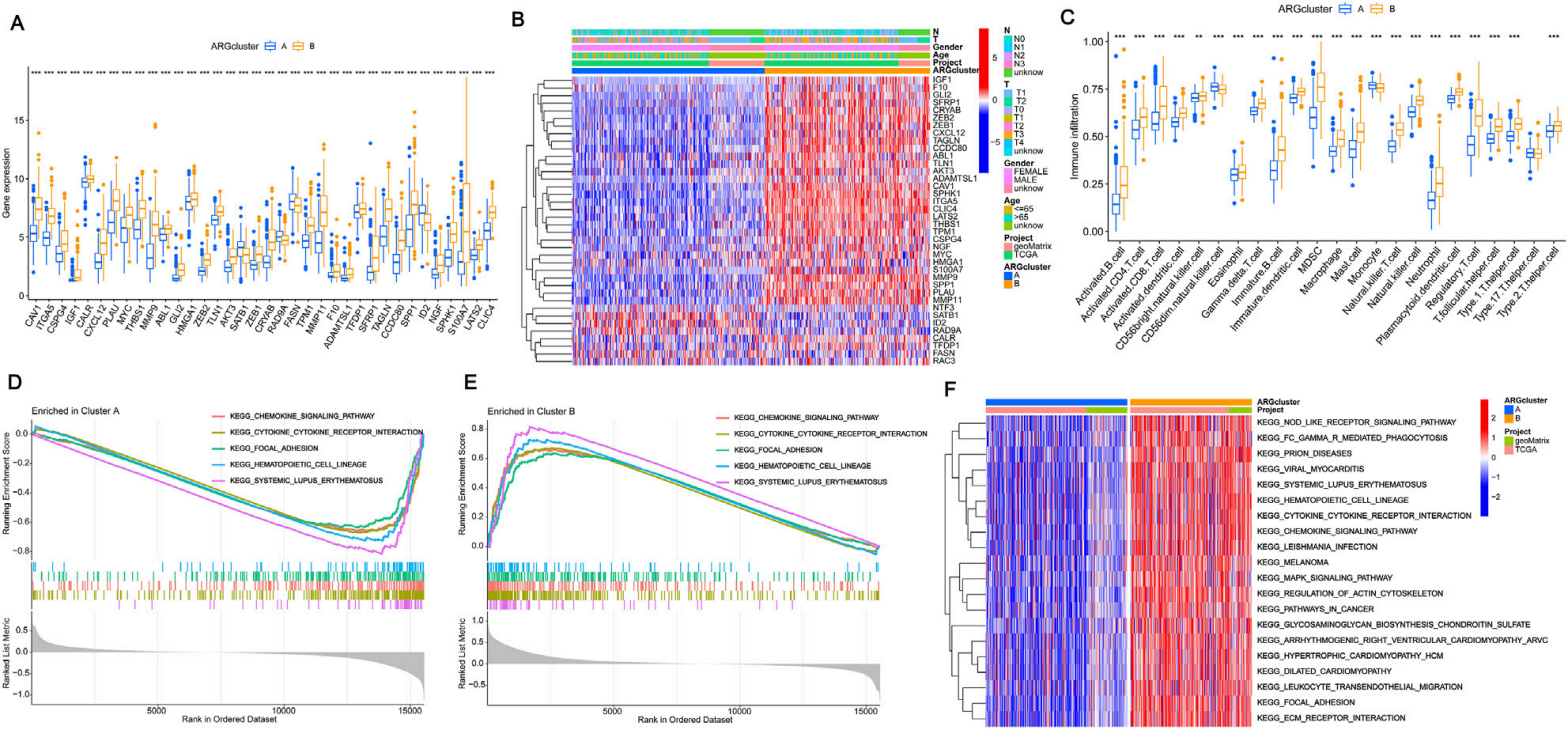
Consensus clustering and immune microenvironment landscape analysis **(A)** Expression of prognostic genes in the two subtype clusters. **(B)** Heatmap of two subtype clusters with prognostic expression and corresponding pathological characteristics (age, sex, N and T stage). **(C)** The variations in immune cell infiltration expression among ARG clusters A and **(B) (D–E)** Cluster B was the main focus of GSEA, with an emphasis on enriching KEGG pathways. **(F)** The Gene Set Variation Analysis of differentially expressed genes identified potential mechanisms underlying the two patterns associated with anoikis. The significance levels were set as *p < 0.05, **p < 0.01, and ***p < 0.001.

To assess the survival of BLCA in different clustering models, The BLCA patients were divided into various subgroups through consensus clustering analysis. The heatmap indicated that K = 2 was the optimal classification for BLCA patients (Figures 2A–C). Cluster A had 204 samples, while Cluster B had 200 samples. Upon analysis of the survival charts, it was determined that Cluster B had a shorter survival time compared to Cluster A (Figure 2D). After conducting principal component analysis (PCA) on the data, it was found that there were significant differences between the clusters (Figure 2E). In addition, we can also distinguish the two clusters through tSNE and UAMP analysis. These results indicate significant differences between the types clustered by these genes (Figures 2F, G). According to the differential analysis, it was found that there was an obvious difference in the expression of the 38 genes between Cluster A and B. The majority of these genes exhibited high levels of expression within Cluster B. However, SATB1, RAD9A, FASN, and ID2 were lowly expressed in Cluster B (Figure 3A), which was borne out by the heatmap (Figure 3B). It can be seen that there are significant differences between the classifications based on these gene clusters, which is very important for classifying the gene expression of different patients so that different treatment methods can be adopted for different types of patients. After analyzing the two clusters, we found variations in immune cell populations. The single-sample gene set enrichment analysis (ssGSEA) results suggested that 22 immune cells exhibited obvious differences between the two subgroups (Figure 3C). According to the Gene Set Enrichment Analysis (GSEA) pathway analysis, Cluster B exhibited high expression levels in all pathways (Figures 3D, E). The Gene Set Variation Analysis (GSVA) revealed distinct variations in KEGG pathways between two clusters, with cluster B showing a clear definition and cluster A showing less description (Figure 3F). These results indicate that there is a connection between these genes and the above pathways, which may provide a potential basis for exploring the pathogenesis of BLCA.

### 3.4 Construction of the prognostic model

**FIGURE 4:**
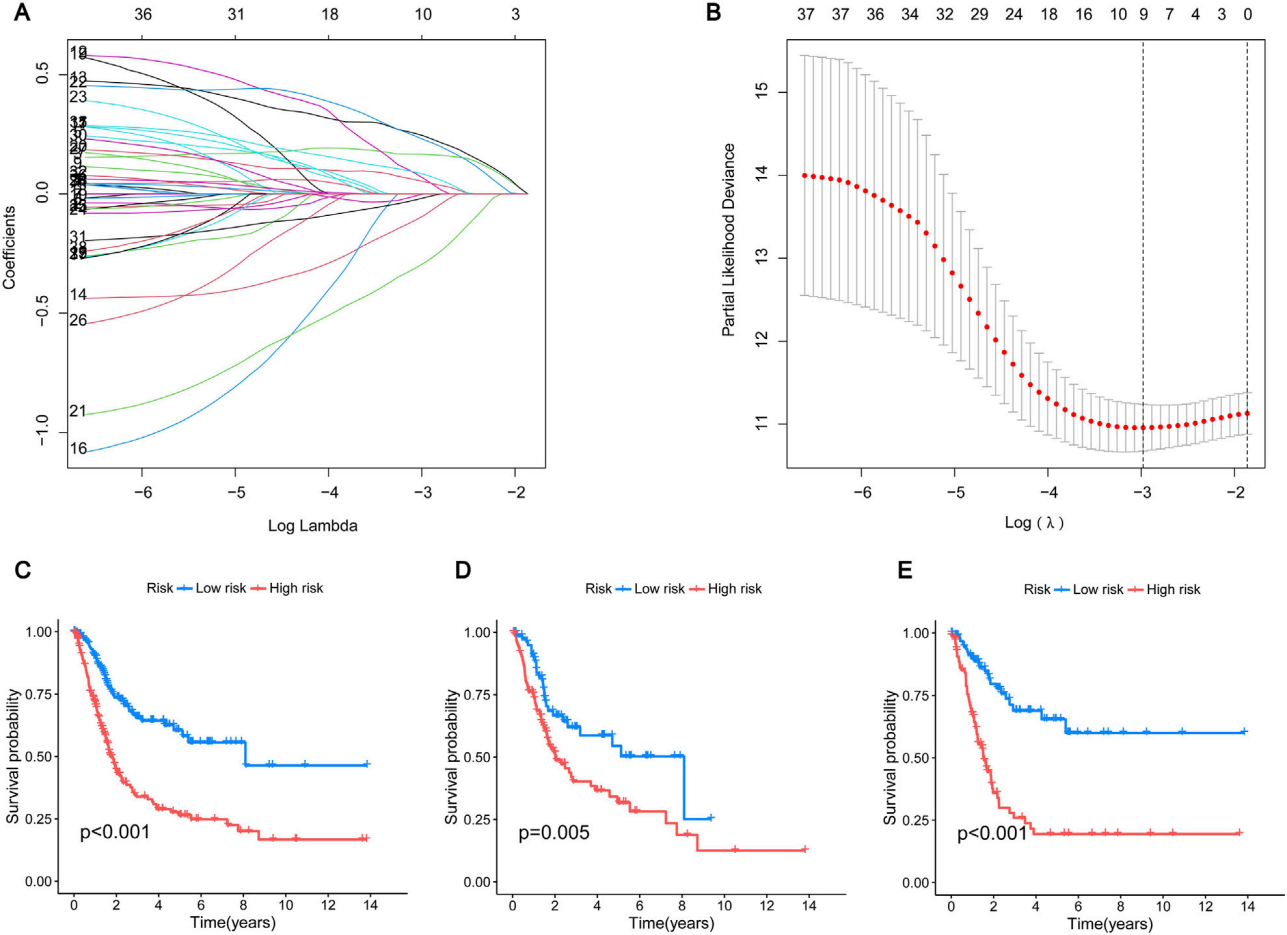
Construction of prognostic model **(A)** The LASSO coefficient of the ARGs in the signature. **(B)** The optimal variable (λ) for the LASSO Cox regression model was chosen using minimal criterion. **(C–E)** Kaplan-Meier survival curves show the change in survival probability in high-risk and low-risk BLCA patients. **(C)** All group; **(D)** Testing group; **(E)** Training group. Blue represents the low-risk group and red represents the high-risk group.

**FIGURE 5:**
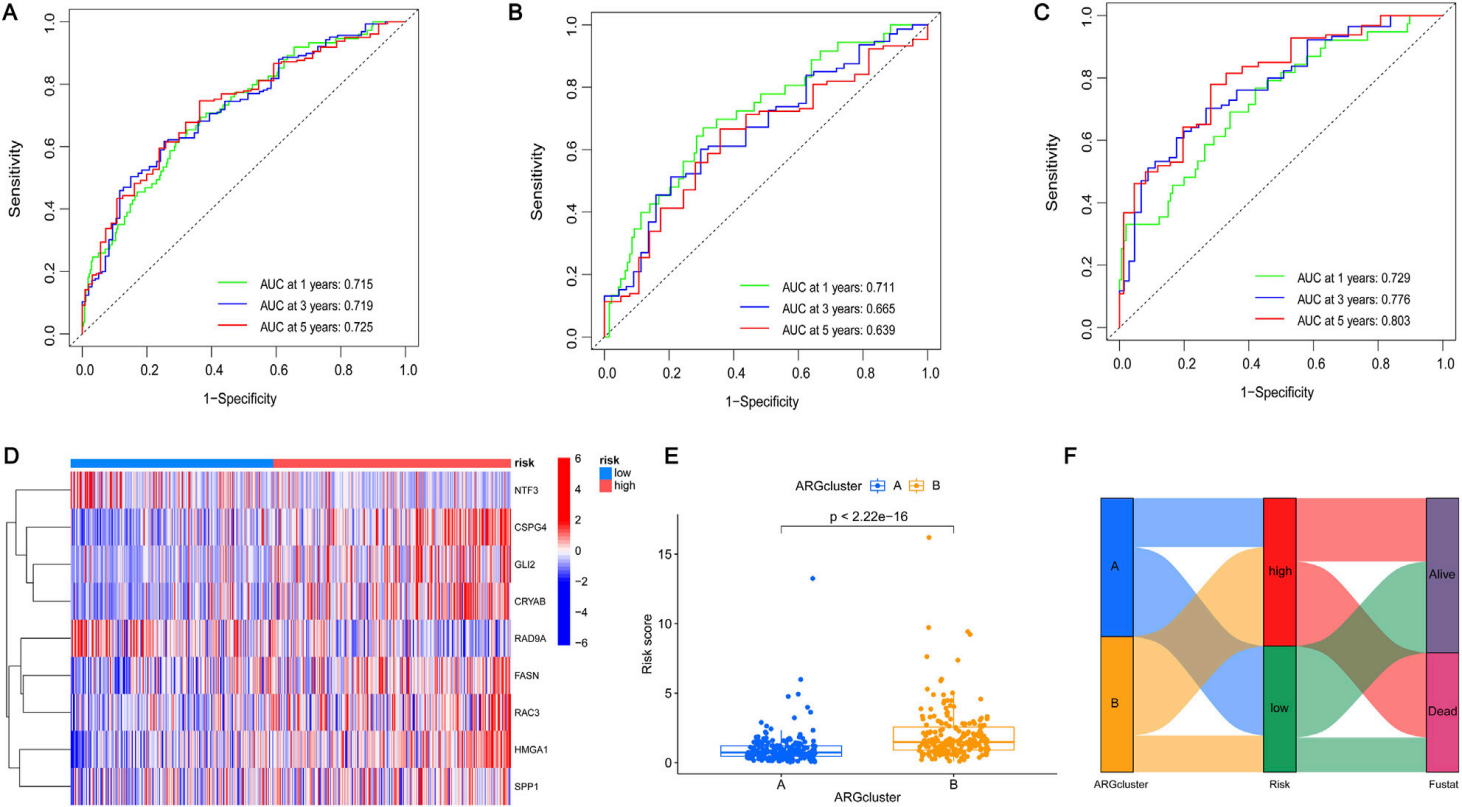
Construction of prognostic model **(A–C)** ROC curves of OS changed over time at 1, 3, and 5 years. **(A)** All group; **(B)** Testing group; **(C)** Training group. The area enclosed by the green, blue, and red lines represents the AUC values in 1, 3, and 5 years respectively. **(D)** Heatmap expression of the prognostic genes in the different risk groups. Blue represents the low-risk group and pink represents the high-risk group. **(E)** Differences in the risk scores between the two subgroups. The *x*-axis represents the type of subgroup, and the *y*-axis represents the risk score. Statistical analysis was performed using the Student’s *T*-test. *p < 0.05, **p < 0.01, and ***p < 0.001. **(F)** Sankey plot of two subgroups, two risk groups, and two clinical outcomes. Genetic clustering was performed on subgroups A and B, classified according to two risk levels (high risk or low risk) and two clinical outcomes (alive or dead).

To further screen out genes with prognostic value, nine genes (CSPG4, GLI2, HMGA1, NTF3, CRYAB, RAD9A, FASN, SPP1, RAC3) were shown to have the best multivariate results and the highest predictive value by LASSO regression analysis (Figures 4A, B). The risk score formula was created using the risk scores of nine genes, calculated as follows: risk score = (CSPG4×0.190927502473781) + (GLI2×0.281101473536596) + (HMGA1×0.356350705812734) - (NTF3×0.429163094370077) + (CRYAB×0.155793970084206) - (RAD9A×0.558367444114141) + (FASN×0.380084107641552) - (SPP1×0.100034977690286) + (RAC3×0.180416238704028). More details can be found in Supplementary Table S5. Patients diagnosed with BLCA were randomly assigned to either the test or training groups. Subsequently, they were categorized as low-risk or high-risk based on their median risk scores for further analysis of their survival rates. The study findings indicated that individuals classified as low-risk demonstrated significantly higher survival rates compared to those categorized as high-risk, highlighting the potential impact of risk stratification on patient outcomes. This suggests that effective risk assessment can play a crucial role in tailoring treatment strategies and improving overall survival in affected populations. This indicates that the created model may be able to differentiate patients more accurately into high-risk and low-risk categories (Figures 4C–E). The ROC curves indicated that the area under the curve for survival rates at 1, 3, and 5 years was greater than 0.6, indicating positive predictive outcomes. The AUC values for patient survival at 1, 3, and 5 years were 0.715, 0.719, and 0.725 across all groups (Figure 5A), 0.711, 0.665, and 0.639 in the test group (Figure 5B), and 0.729, 0.776, and 0.803 in the training group (Figure 5C), suggesting that the model can reliably forecast patient survival, thus confirming the credibility of the model. The risk heatmap illustrated gene expression levels across high-risk and low-risk categories, revealing that, with the exception of NTF3 and RAD9A, the other seven genes exhibited significantly elevated expression levels in the high-risk group (Figure 5D). This pattern underscores the potential role of these genes in contributing to the increased risk profile, suggesting their relevance as biomarkers for risk assessment and targeted therapeutic strategies. We conducted an analysis to determine if there is any relationship between consensus clustering and risk scores. The results show a notable disparity in risk scores between the two categories, with group B exhibiting notably higher risk scores compared to group A (Figure 5E). The Sankey diagram distinctly shows the association between clusters and risk scores associated with anoikis (Figure 5F). These results indicate that the risk model constructed based on these 9 genes can accurately predict the prognosis of BLCA patients.

### 3.5 Construction of a nomogram

**FIGURE 6:**
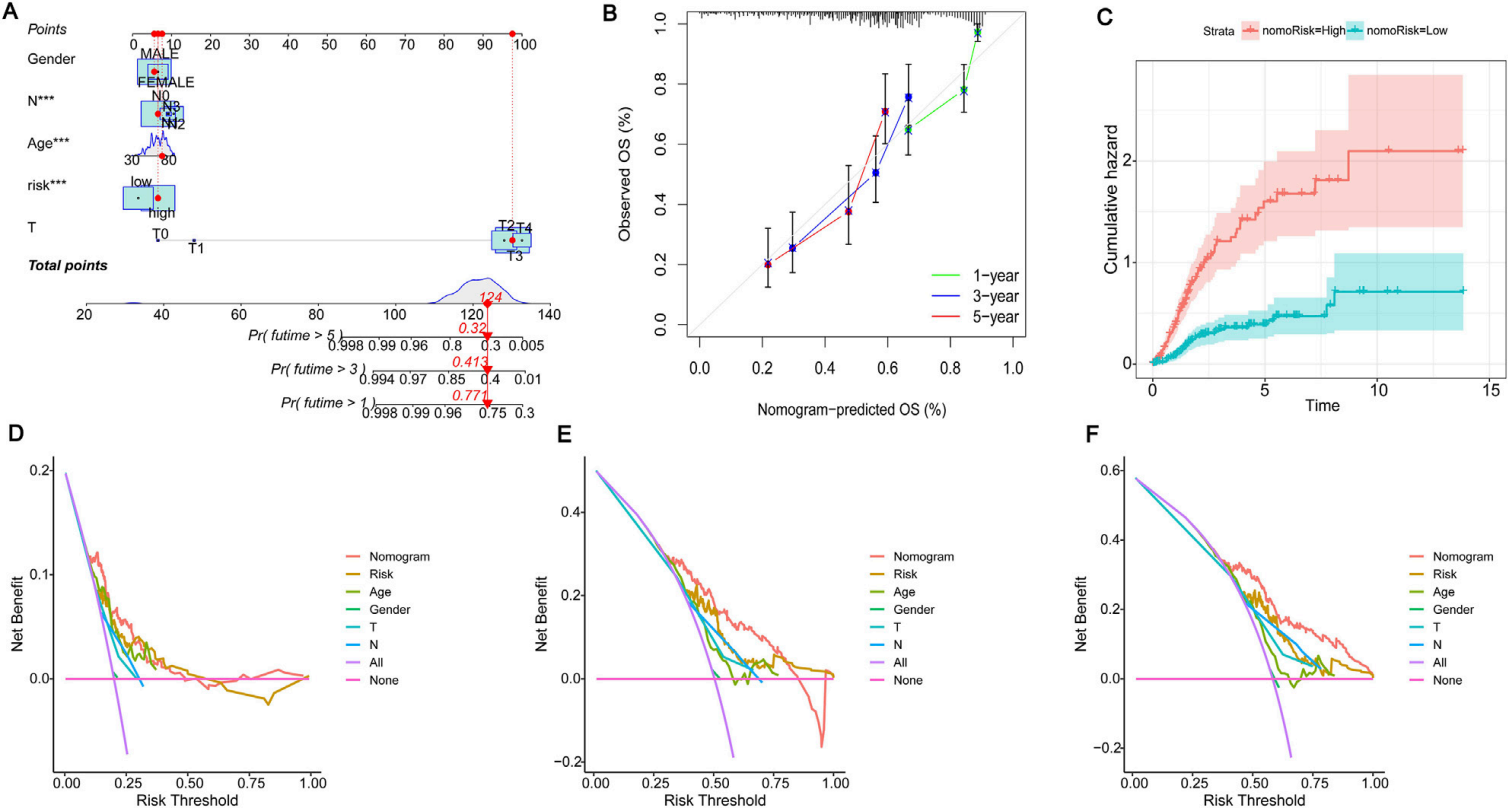
Construction of the nomogram. **(A)** The nomogram for the prediction of overall survival in BLCA based on the risk score. “Points” is a scoring scale for individual factors. “Total Points” is the sum of the scoring scale for each factor, such as risk score, age, gender, and stage. The overall survival rate of 1–5 years was inferred according to “Total Points”. **(B)** Calibration plots to validate the nomogram. **(C)** Cumulative hazard curves represented the probability of survival over time progression. **(D–F)** Decision curve analysis (DCA) curves of 1-, 3-, and 5-year OS column line graphs for BLCA patients.

Due to the significant importance of risk scores in evaluating patient outcomes, we plan to combine genetic risk scores with other clinicopathologic elements to establish a nomogram that can predict the chances of survival for individuals with BLCA. To assess the survival of BLCA patients, a score is assigned to each clinical characteristic. These scores are then combined to calculate the total score. Figure 6A displays a total score of 124 for the patient, suggesting survival rates of 0.771, 0.413, and 0.32 at 1, 3, and 5 years, respectively. The projected calibration graph showed a strong association with the 1-, 3-, and 5-year overall survival (OS) curves in contrast to the perfect model for the entire group (Figure 6B). Risk for patients escalated with time and was notably greater for those in the high-risk category in comparison with those in the low-risk category (Figure 6C). Decision curves showed that the constructed column-line graphs better predict patient survival (Figures 6D–F). These results further demonstrate that the risk model we constructed can accurately predict the prognosis of BLCA patients.

### 3.6 Immune microenvironment analysis and screening potential therapeutic drugs

**FIGURE 7:**
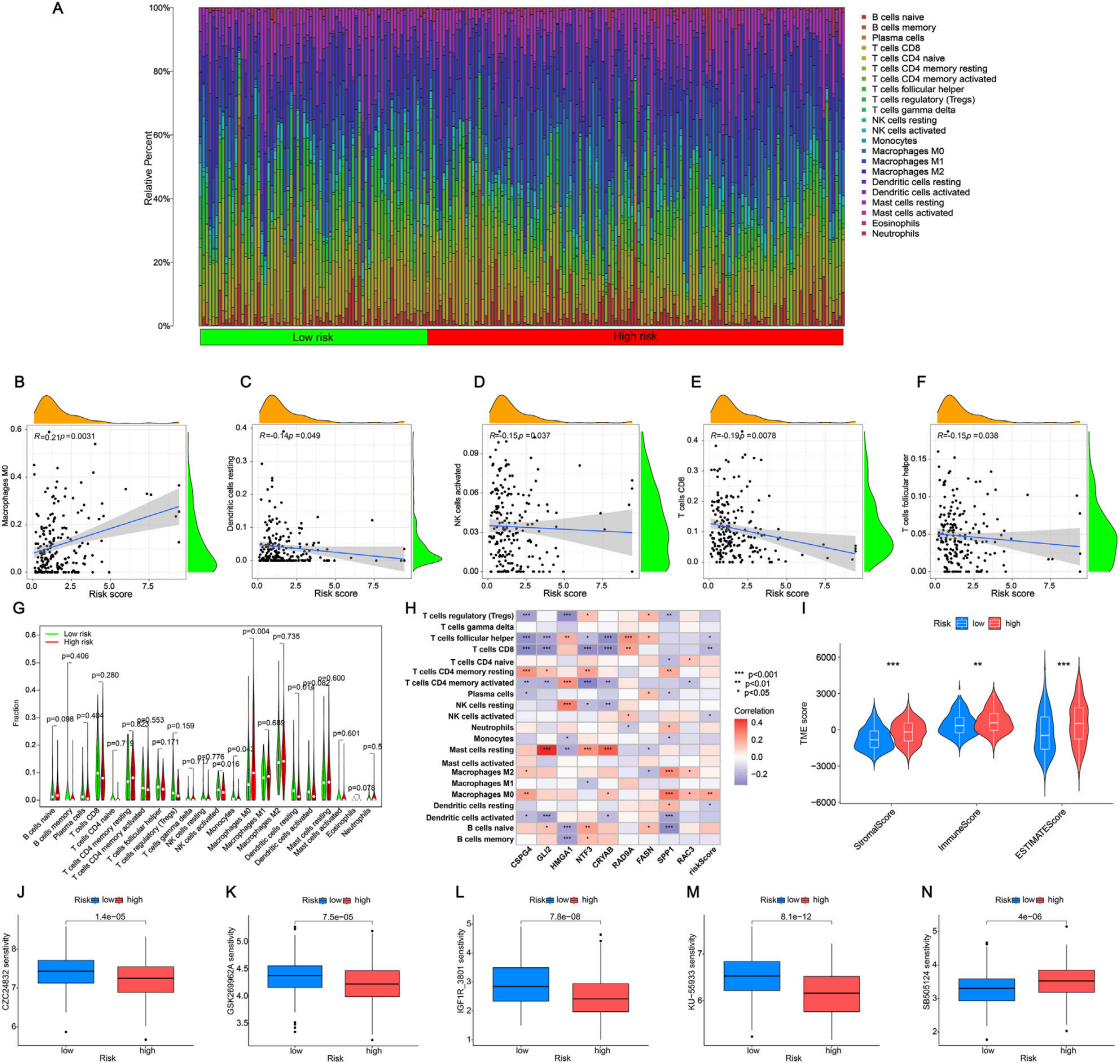
Analysis of immune cell infiltration in the tumor microenvironment **(A)** Individual BLCA patients’ respective levels of immune cell infiltration. **(B–F)** The relationships between the risk score and the infiltration level of immune cells. **(G)** The violin plot compares the immune cells’ infiltration levels between low-risk and high-risk categories. **(H)** Correlation of nine ARGs with immune cells. Red represents a positive correlation, blue represents a negative correlation, and the darker the color, the stronger the correlation. **(I)** The immune, stromal, and ESTIMATE scores were calculated using the ESTIMATE method and compared between the two groups. **(J–M)** Drugs are susceptible to low-risk groups (partial). **(N)** Drugs are susceptible to high-risk groups. The significance levels were set as *p < 0.05, **p < 0.01, and ***p < 0.001.

We analyzed the discrepancies in immune cell populations between the two subgroups since the immune response is crucial to fight cancer. The level of immune cell infiltration differed significantly between the high and low-risk categories (Figure 7A). Additionally, the correlation heatmap illustrated the relationships between various immune cells, with stronger colors and higher values indicating better correlation (Supplementary Figure S4). Figures 7B–F displayed the importance of various immune cells (Macrophages M0, resting Dendritic cells, CD8^+^ T cells, follicular helper T cells, and activated NK cells) in distinguishing between the two groups. Upon further analysis, only four immune cells exhibited obvious discrepancies in risk scores between the high-risk and low-risk groups. Specifically, Macrophages M0 were highly expressed in the high-risk group, while NK cells activated, Monocytes, and resting Dendritic cells were highly expressed in the low-risk group (Figure 7G, *p* < 0.05). As shown in Figure 7H there was a specific correlation between 9 ARGs (CSPG4, GLI2, HMGA1, NTF3, CRYAB, RAD9A, FASN, SPP1, RAC3) and immune cells. Statistically significant differences in StromalScore were found between the high-risk and low-risk groups. The high-risk group had a significantly higher TME score than the low-risk group (Figure 7I). There are different immune statuses between different risk models, indicating that the model we built can participate in the immune response of BLCA patients, which can help doctors better predict the patient’s survival and recurrence risk.

Medication is a crucial component of the cancer treatment journey. Hence, we will assess the responsiveness of BLCA patients to medications linked with prognostic genes, revealing a notable variation in drug sensitivity among 60 medications between the high-risk and low-risk categories. In the high-risk group, drugs such as Aliseritib and AZD536 demonstrated significantly higher half-maximal inhibitory concentration (IC50) values, indicating a low sensitivity to these medications (Figure 7J–M). Notably, only SB505124 medications exhibited significantly elevated levels in the high-risk category, indicating enhanced sensitivity of the medication in the high-risk group compared to the low-risk group (Figure 7N). Additional information can be found in Supplementary Table S6. The findings above indicate that these medications may serve as promising treatment options for BLCA chemotherapy, potentially enhancing the drugs’ efficacy and usage.

### 3.7 Screening for separate predictive genes and initial validation

**FIGURE 8:**
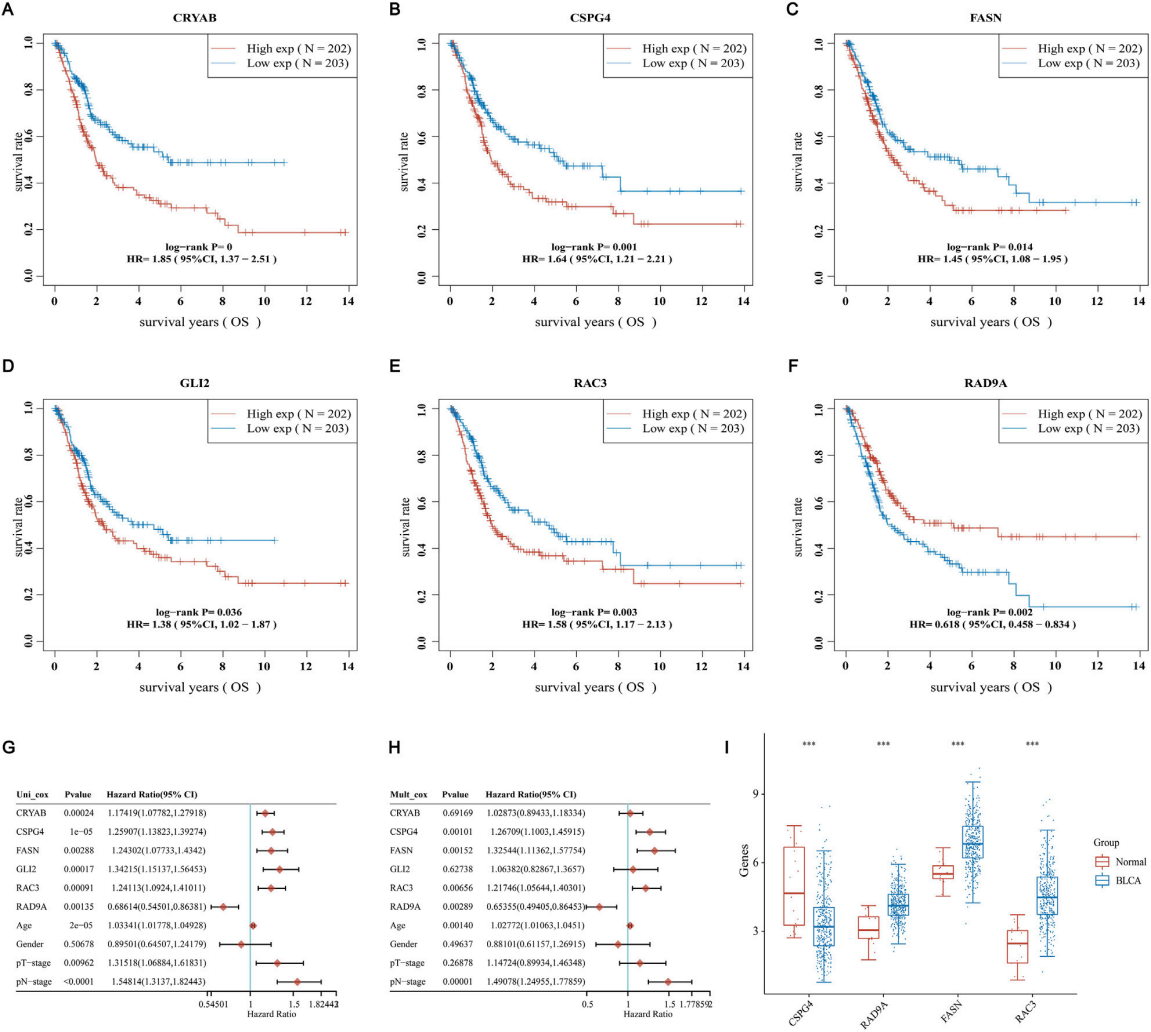
Screening for independent prognostic genes **(A–F)** Comparison of OS of six genes in BLCA high and low expression groups. **(G, H)** Hazard ratio and *p*-value of the constituents involved in univariate and multivariate Cox regression considering clinical parameters and six prognostic ARGs in BLCA. **(I)** Comparison of the expression of these genes in BLCA tissues and adjacent normal tissues.

To investigate ARGs’ role in BLCA further, we first validated the survival status of these nine genes. We found that six genes had significant differences in the survival time of BLCA. All of them had lower survival rates with survival time, and the high-expression groups of CRYAB, CSPG4, FASN, GLI2, and RAC3 had lower survival rates than those of the common expression ones. In contrast, the RAD9A high-expression group had a greater survival rate in comparison with the low-expression group (Figures 8A–F). Along with that, univariate and multivariate Cox regressions using these six genes and age, gender, pT, and pM staging revealed that CSPG4, RAD9A, FASN, and RAC3 could be used as independent prognostic genes in BLCA (Figures 8G, H). Afterward, an analysis was conducted to compare the expression of four genes in both BLCA and adjacent normal tissues. The results showed that only CSPG4 was expressed at low levels in the tumor tissues, while the other three genes exhibited high levels of expression in the tumor tissues (Figure 8I). Since the survival and differential expression analysis results showed that both FASN and RAC3 were highly expressed in tumor tissues, the next step was carried out.

### 3.8 Validation of RAC3 expression and prognostic function in BLCA

**FIGURE 9:**
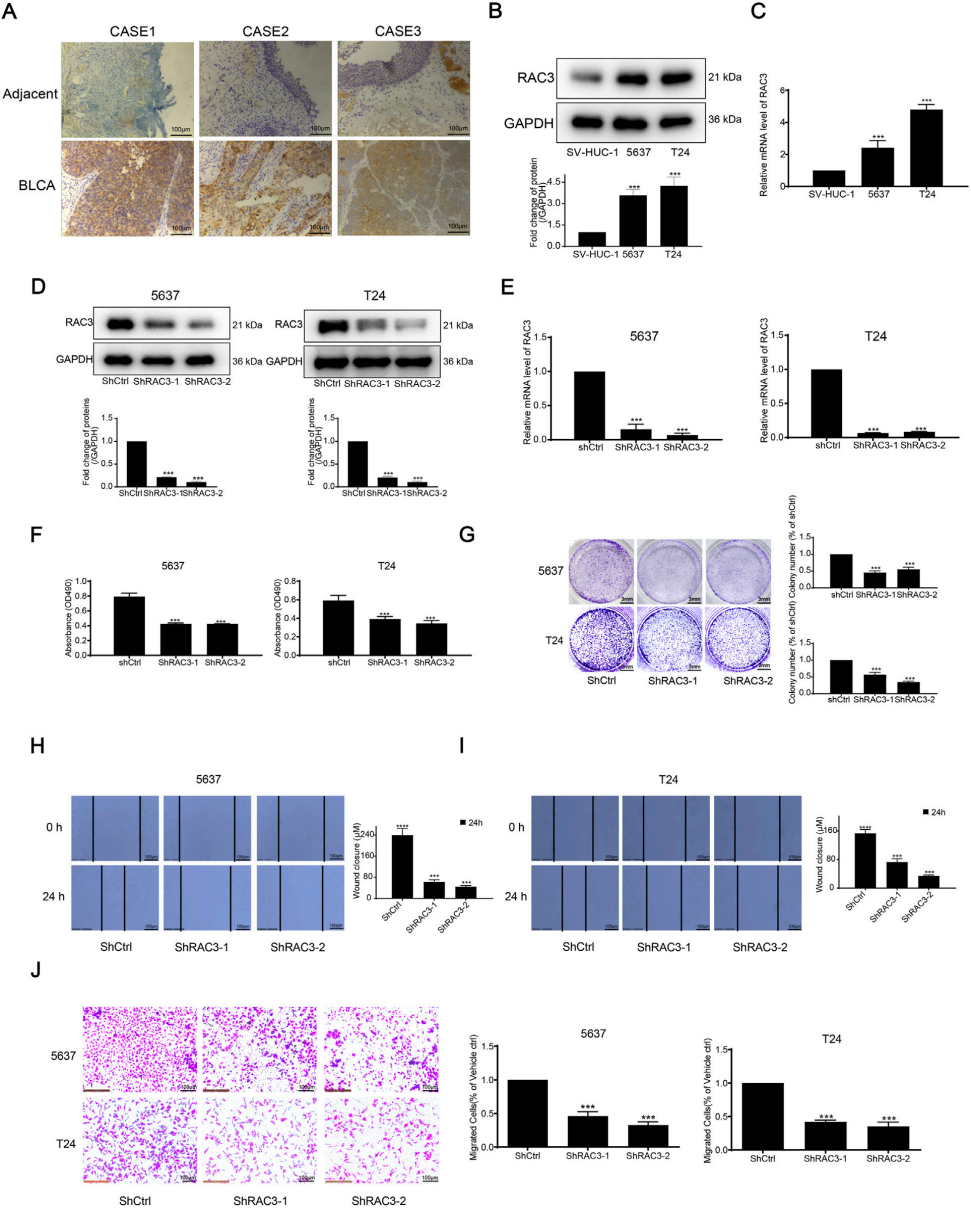
RAC3 is overexpressed in BLCA tissue and cells and validation of RAC3 prognostic function in BLCA **(A)** IHC determined the expression of RAC3 in BLCA and adjacent tissues. **(B)** The expression of RAC3 in two BLCA cells and one normal bladder cell was determined by WB. **(C)** The expression of RAC3 in two BLCA cells and one normal bladder cell was determined by qPCR. **(D)** The expression of RAC3 after silencing RAC3 was measured by WB. **(E)** The expression of RAC3 after silencing RAC3 was measured by qPCR. **(F)** After silencing RAC3, cells were inserted into the 96-well plate. After another 72 h, the proliferation of cells was detected by MTT. **(G)** After silencing RAC3, cells were inserted into the 24-well plate. After another 6–8 days, colony suppression after silencing RAC3 was evaluated by clonogenic assay. **(H–J)** After silencing RAC3, cells were inserted into 12-well plate or transwell chamber. Scratch and transwell assay was used to evaluate migration suppression after silencing RAC3.

Analysis of previous literature revealed that there have been reports of FASN studies in BLCA (Dong et al., 2023), so in this study, we decided to select RAC3 for *in vitro* validation of BLCA. We stained three neighboring tissues and three BLCA tissues with immunohistochemistry to look into the expression of RAC3 in BLCA. IHC labeling revealed that RAC3 was more abundant in BLCA than in nearby tissues (Figure 9A). Then, we assessed RAC3 expressions in two BLCA and one normal bladder cell line using WB and qPCR methods. Figures 9B, C presented our findings, which showed that RAC3 protein and mRNA levels were considerably more significant in BLCA cells than in normal cells. We thus investigated RAC3’s function in BLCA in further detail. After confirming that RAC3 was successfully silenced using WB and qPCR testing (Figures 9D, E), we carried out several experiments to find out how RAC3 affected BLCA, including MTT, clonogenic, scratch, and transwell assays. According to the findings, RAC3 silencing dramatically reduced cell migration, clonogenesis, and proliferation (Figures 9F–J). These outcomes collectively suggest that RAC3 plays a vital part in the migration and proliferation of BLCA. Interestingly, results from co-incubation of human BLCA cells with CD8^+^ T cells showed that these two cancer cell lines could be killed by activated T cells more easily when RAC3 was absent (Supplementary Figure S5A). The findings emphasize the crucial role of RAC3 in the proliferation, metastasis, and immune escape of BLCA cells. We have already screened SB505124 as a potential therapeutic agent for BLCA. Therefore, we proceeded to conduct further investigations on the *in vitro* antitumor activity of SB505124. Supplementary Figure S5B demonstrates that SB505124 exhibited a significant inhibitory effect on the proliferation of BLCA cells. These findings strongly suggest that SB505124 holds promise as a potential treatment option for BLCA.

### 3.9 RAC3 knockdown inhibited the growth and migration of BLCA cells *in vivo*

**FIGURE 10:**
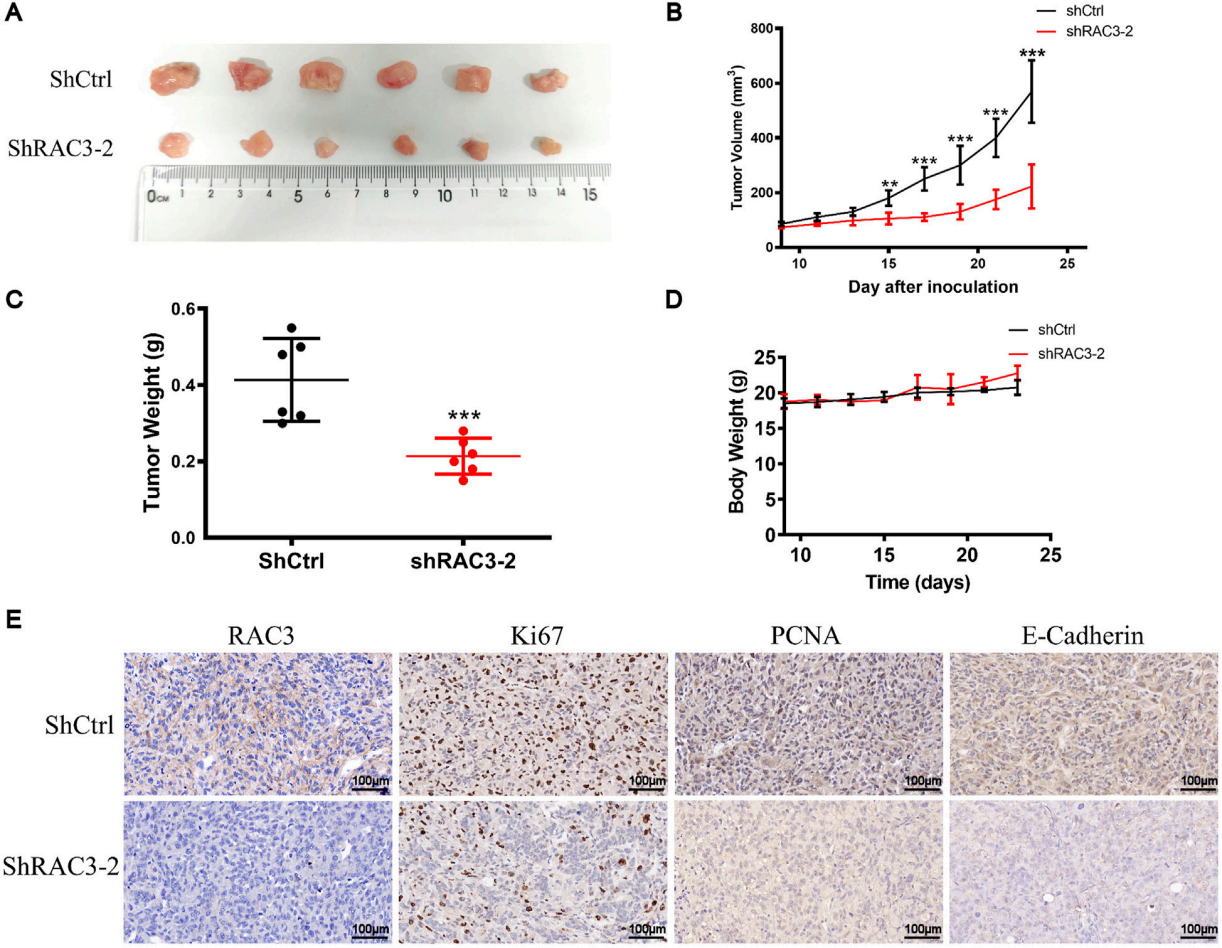
RAC3 knockdown inhibited the growth and migration of BLCA cells *in vivo*. **(A, B)** 5 × 10^6^ T^24^-shCtrl and T24-shRAC3-2 cell suspensions were injected into the right flank of mice. When the tumor volume reached 70–100 mm^3^ (9 days after cells were injected into the mice flank), each mouse’s tumor volume was measured every 2 days. 23 days after cells were injected into the flank of mice, tumors were removed and photographed. **(C)** Statistical analysis of tumor weight at 23 days **(D)** Changes in each group mice weight. **(E)** The expression of RAC3, Ki67, PCNA, and E-Cadherin in tumor tissues (ShCtrl and ShRAC3-2 group) was detected by IHC. The plotting scale is 100 μm.

We further explored the impact of RAC3 knockdown on tumor growth *in vivo* using a subcutaneous xenograft model in nude mice. Cells expressing shRAC3-2 and non-targeting control shCtrl were injected, and the results demonstrated significant inhibition of tumor growth upon RAC3 knockdown. The tumor growth inhibition rates were measured as 61% (Figures 10A, B). Furthermore, the shRAC3-2 group exhibited a 49% decrease in tumor weight compared to the shCtrl group (Figure 10C). Notably, the average body weight of the mice remained unaffected by RAC3 knockdown (Figure 10D). Immunohistochemical findings indicated a notable decrease in the quantity of Ki-67 and PCNA-positive cancer cells in the shRAC3-2 group when compared to the shCtrl group, as demonstrated in Figure 10E. Additionally, immunohistochemistry results confirmed that RAC3 knockdown downregulated the expression of the migration-related protein E-Cadherin *in vivo* (Figure 10E). These outcomes strongly sustain the idea that RAC3 knockdown hinders the growth and movement of BLCA cells *in vivo* by reducing the expression of Ki67, PCNA, and E-Cadherin.

## 4 Discussion

BLCA exhibits a low 5-year survival rate and a grim prognosis. Consequently, improving the prognosis and extending the patients’ survival time depends heavily on early diagnosis, prompt treatment, and risk assessment of BLCA patients.

By comparing the expression of ARGs in BLCA tissues and normal tissues, we studied how they affect the tumor microenvironment. A variety of immune cells are involved in the development of BLCA. Macrophages are innate immune cells widely distributed in the body and play a central role in maintaining the body’s immune homeostasis and eliminating pathogens. M0 macrophages are undetermined cells that can polarize into M1 and M2 macrophage subpopulations when disturbed by different factors. The two play different roles in the tumor immune microenvironment. M1 macrophages help enhance anti-tumor immunity, while M2 macrophages contribute to tumor growth and metastasis (Wu J. et al., 2024). Therefore, promoting the polarization of M0 macrophages toward M1 and inhibiting M2 polarization seems to control the growth and metastasis of tumor cells. Studies have found that interfering with SLC3A2 can inhibit the M2 polarization of BLCA tumor-associated macrophages, thereby inhibiting BLCA cell proliferation, invasion, and migration (Wu P. et al., 2024). CD8^+^ T cells are a subset of T cells that can specifically recognize and kill cancer cells. Based on CD8^+^ T cells, immunotherapy of tumors can be carried out (Sun et al., 2023). Some studies have found that S100A5 weakens the killing of BLCA cells by effector T cells by inhibiting CD8^+^ T cell proliferation and cytotoxicity, thereby exerting anti-cancer immunity and further reducing the effectiveness of anti-PD-L1/PD-1 immunotherapy (Li et al., 2023). Therefore, identifying immune cells with significant associations with risk genes can help us understand tumor cell immune evasion, microenvironmental changes, and response to treatment. It can provide new ideas for early treatment and personalized treatment of patients. On this basis, we conducted *in vitro* and animal experiments to prove the key role played by RAC3 in the migration and proliferation of BLCA. In previous research, this was found. RAC3 is a tumor-promoting factor that can promote the proliferation, migration, and invasion of BLCA, and may be related to the activation of the PYCR1/JAK/STAT signaling pathway (Cheng et al., 2020). In our study, we found that the proliferation, migration, and invasion of BLCA were inhibited by knocking down the expression of RAC3. Some studies have confirmed our results. Liwei Wang’s team found that high expression of RAC3 is negatively correlated with the prognosis of BLCA patients. Its knockdown will lead to cell cycle arrest and reduced adhesion and can activate PI3K/AKT/mTOR-mediated autophagy and inhibit BLCA cell proliferation and migration *in vivo* and *in vitro* (Wang et al., 2022). In addition, many signaling pathways are involved in RAC3 regulating the proliferation and migration of BLCA, such as cytokine-cytokine receptor interaction, hematopoietic cell lineage, etc. These differ in the two-cluster classification, so they may also be involved in RAC3 regulation. BLCA is functional, but further research is needed to verify it.

After analyzing the existing literature, a study was conducted to establish non-invasive BLCA using a mouse bladder model and treating the tumor with GLI2ASO. The results indicated a decrease in tumor size, growth rate, GLI2 messenger RNA, and protein expression, indicating that GLI2ASO may serve as a specific treatment for BLCA with promise for advancement (Raven et al., 2019). Certain research has indicated that miR-15a-5p is directed towards HMGA1, while LINC00649 boosts HMGA1 levels by attaching to miR-15a-5p. Also, heightened levels of HMGA1 have been shown to somewhat counteract the growth, movement, and infiltration of BLCA cells (Chen and Chen, 2021). CRYAB, a frequently occurring inhibitor in BLCA, hinders the movement and infiltration of tumor cells by blocking the activation of the PI3K/AKT and ERK signaling pathways, thus impeding tumor cell migration and invasion (Ruan et al., 2020). RAC3, also referred to as RAC-associated C3 botulinum substrate 3, is a member of the Rho family of small GTPases and is found in many different tissues. Currently, many studies have shown that RAC3 may play a vital part in cell adhesion, migration, invasion, and apoptosis in different types of cancers, including breast (Rosenberg et al., 2017) and endometrial cancers (Jin et al., 2023), with anoikis being a crucial factor (Baugher et al., 2005; Didsbury et al., 1989). A study has shown that RAC3 is present in serum and urine samples of patients with chronic cystitis and bladder tumors, but the expression level is significantly higher in bladder tumors. Furthermore, the presence of RAC3 was more evident in the blood and urine of individuals with advanced stages of BLCA (Wang et al., 2024). Further examination uncovered that RAC3 exhibited high levels of expression in BLCA tissues and cells, correlating with a poor prognosis in advanced malignancies. The results showed a distinct process by which BLCA cells migrate and multiply, with RAC3 inhibiting autophagy mediated by PI3K/AKT/mTOR. Treating BLCA patients with co-targeting RAC3 and mTOR will provide new therapeutic options (Wang et al., 2022).

SB505124 is a selective inhibitor of the TGF-β pathway, and the inhibition of this pathway continues to be an active area of cancer research. Within the tumor microenvironment (TME), disrupted TGF-β communication inhibits the body’s ability to fight against tumors and encourages the growth of cancerous fibrosis, the transition of cells from epithelial to mesenchymal, and the formation of new blood vessels (Gulley et al., 2022). Elevated TGF-β levels in the TME correlate with poor clinical outcomes and increased risk of metastasis (Agajanian et al., 2015; Calon et al., 2015). Preclinical research indicates that blocking TGF-β signaling may halt fibrosis, EMT, and angiogenesis while also inhibiting tumor progression (Akhurst and Hata, 2012; Colak and Ten Dijke, 2017). Studies have found that acidic pH promotes autocrine TGF-β2 signaling, which in turn facilitates the formation of lipid droplets (LD), which can be used to support anoikis resistance and energy storage for cancer cell invasiveness, thus expanding the range of cancer cells. Driving role of metabolic adaptations to extend survival in cancer patients (Corbet et al., 2020). Despite this, the correlation between RAC3 and SB505124 is still unclear. This study discovered the potential role between them for the first time. Galunisertib, a TGF-βRI inhibitor, was used alone in translational research to enhance T-cell immunity against tumors and promote antigen spread in a breast cancer mouse model. Galunisertib was given therapeutically to enhance the anti-cancer effect of an anti-PD-L1 treatment on a colorectal cancer mouse model. This was done to promote tumor shrinkage and increase T-cell activity (Holmgaard et al., 2018). Increased levels of TGF-β1 stimulate the growth of tumors and the spread of cancer to the lungs in the later phases of lung cancer development (Wang et al., 2022; Dumont and Arteaga, 2003; Wakefield and Roberts, 2002). TGF-β1 is crucial in the advancement and spread of cancer (Fenteany and Zhu, 2003). The studies above demonstrate the significant role of TGF-β in cancer development. Inhibition of TGF-β could potentially positively impact cancer treatment to some extent. In the drug sensitivity analysis, it was found that SB505124 is highly effective in treating BLCA. Therefore, this drug has the potential to be used as a therapeutic agent for BLCA chemotherapy, and its effectiveness can be further improved in subsequent treatments.

Although our study achieved some promising results, there are still areas where our study could be improved. The BLCA samples used in the study were sourced from public repositories and may lack key details or experience delays in data integration. Some useful genes may not be tested because predictive models are built by evaluating a small number of unique risk genes. Only mice were selected as the experimental subjects in our animal experimental model. There is a lack of other animal models, which may have some selection bias on the results. There are still many challenges in applying our research to clinical practice, and further investigation of our study is necessary.

## 5 Conclusion

The predictive model developed in this study precisely predicts the prognosis of patients with BLCA. SB505124 could become an important drug in the treatment of BLCA patients. RAC3 plays a crucial part in predicting outcomes, the immune environment, and the aggressive nature of BLCA both in experimental settings and living organisms. It will also offer the potential for personalized treatment for BLCA patients and generate new research avenues for clinical investigators.

## Data Availability

The original contributions presented in the study are included in the article/Supplementary Material, further inquiries can be directed to the corresponding authors.
