# Supplementary material for: Role of anoikis-related gene RAC3 in prognosis, immune microenvironment, and contribution to malignant behavior in vitro and in vivo of bladder urothelial carcinoma
Source: Front Pharmacol. 2024 Nov 26;15:1503623. doi: 10.3389/fphar.2024.1503623 (PMC11628291; doi:10.3389/fphar.2024.1503623)
Supplement: Supplementary file 1 [file DataSheet1.docx]

Supplementary Material

**Figure S1**

**
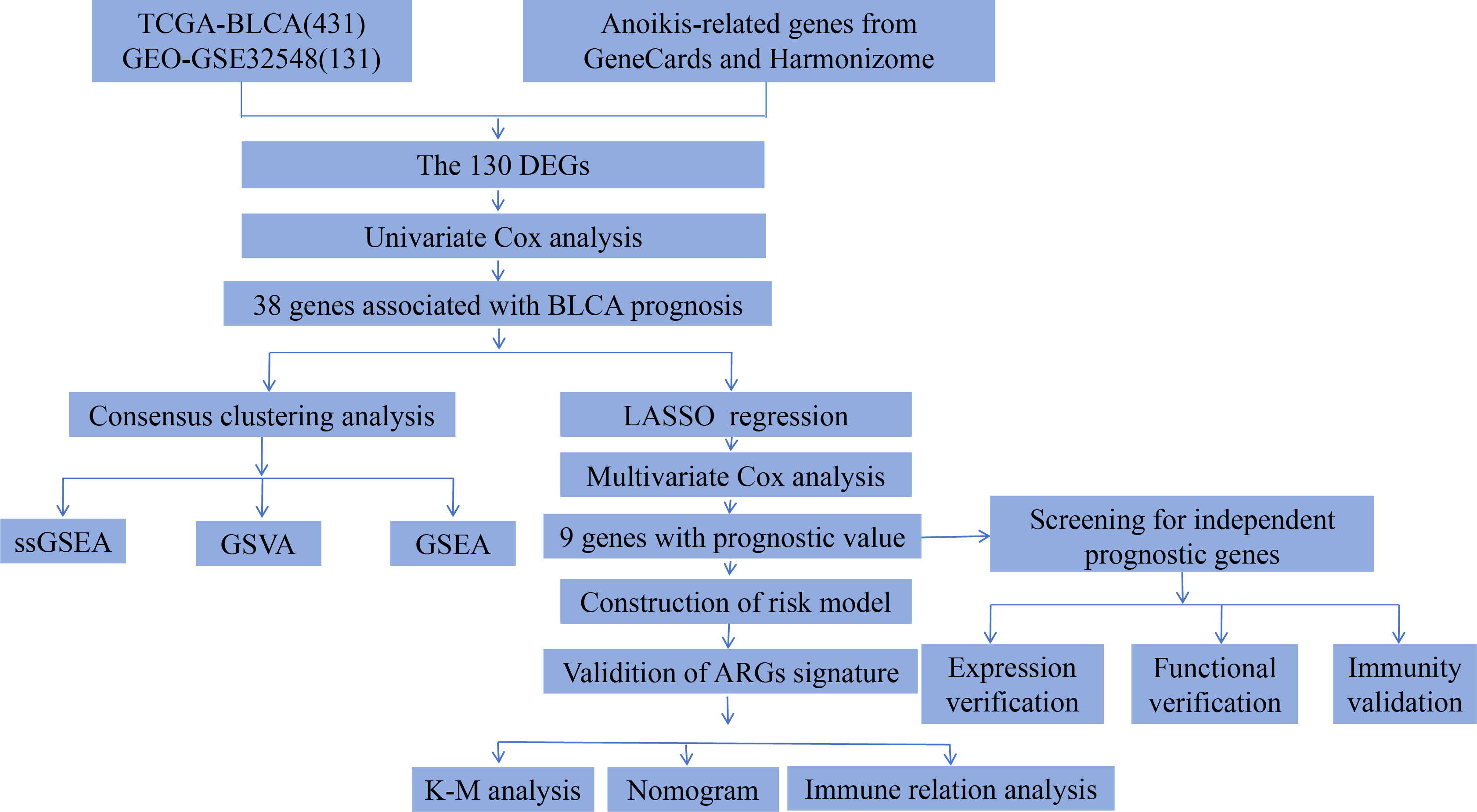
**

**Figure S1.** The workflow of this study.

**Figure S2**

**
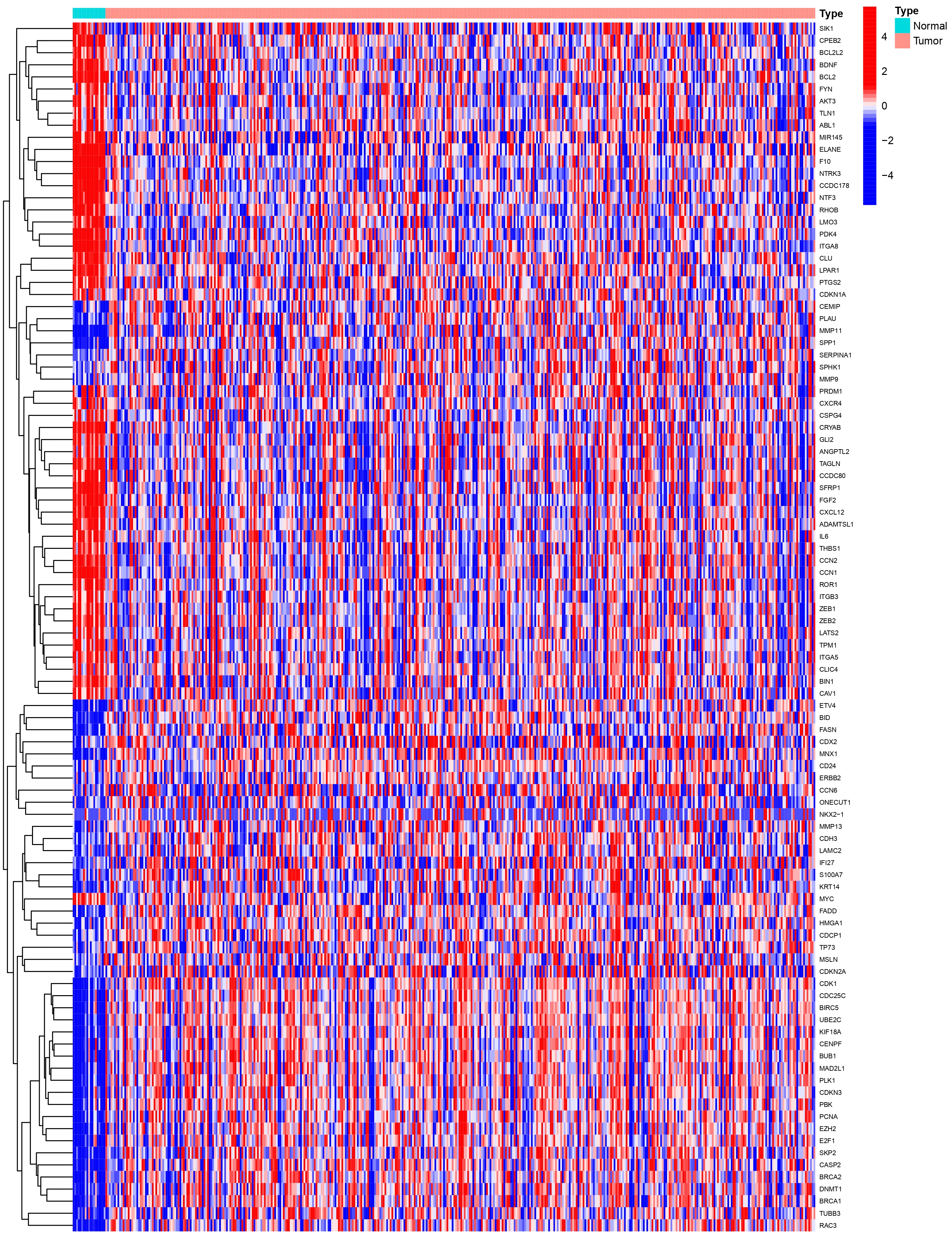
**

**Figure S2.** Heatmap showing differential gene expression in BLCA

**Figure S3.**

**
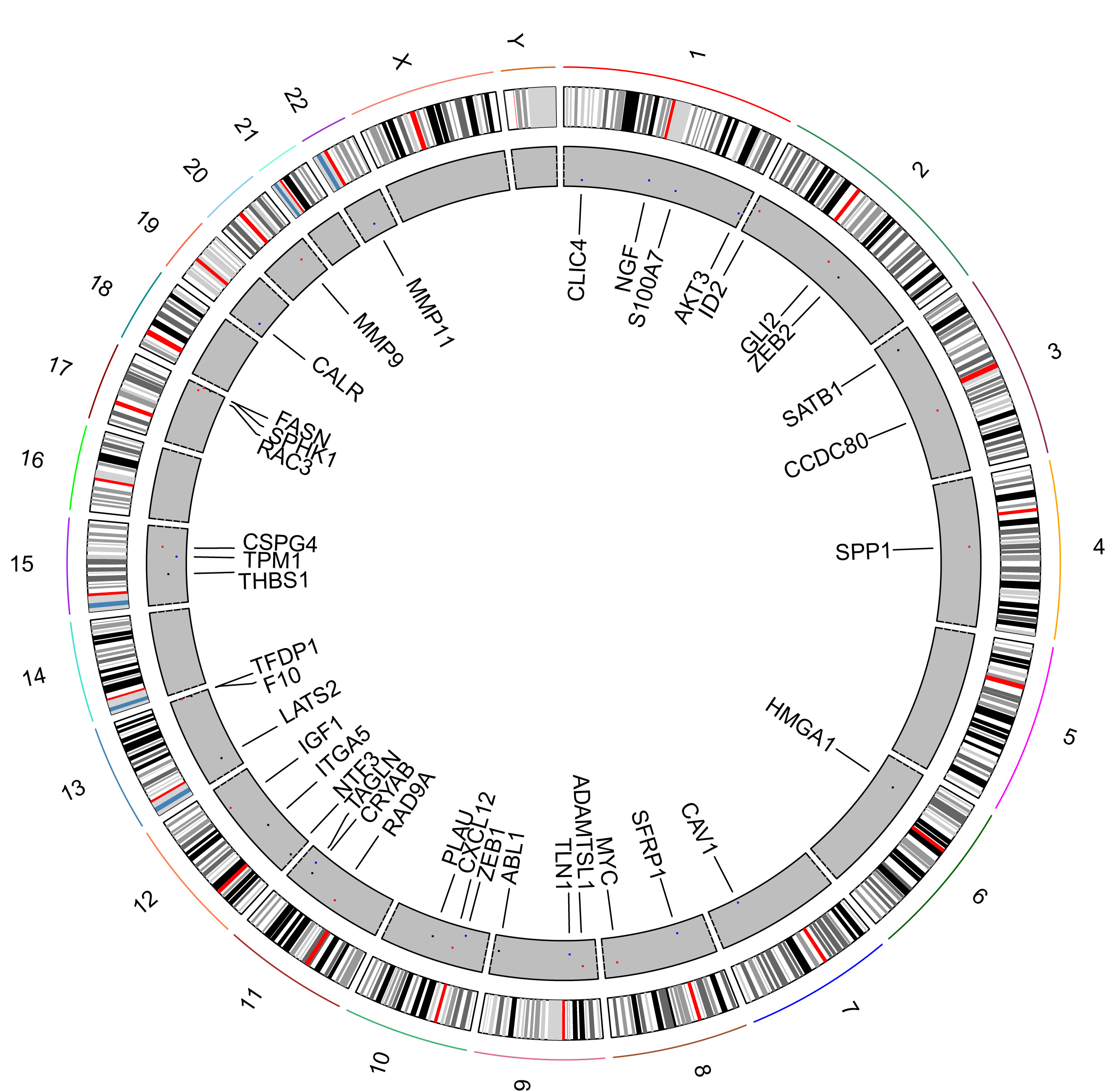
**

**Figure S3.** Copy number circle plot of 38 genes

**Figure S4.**

**
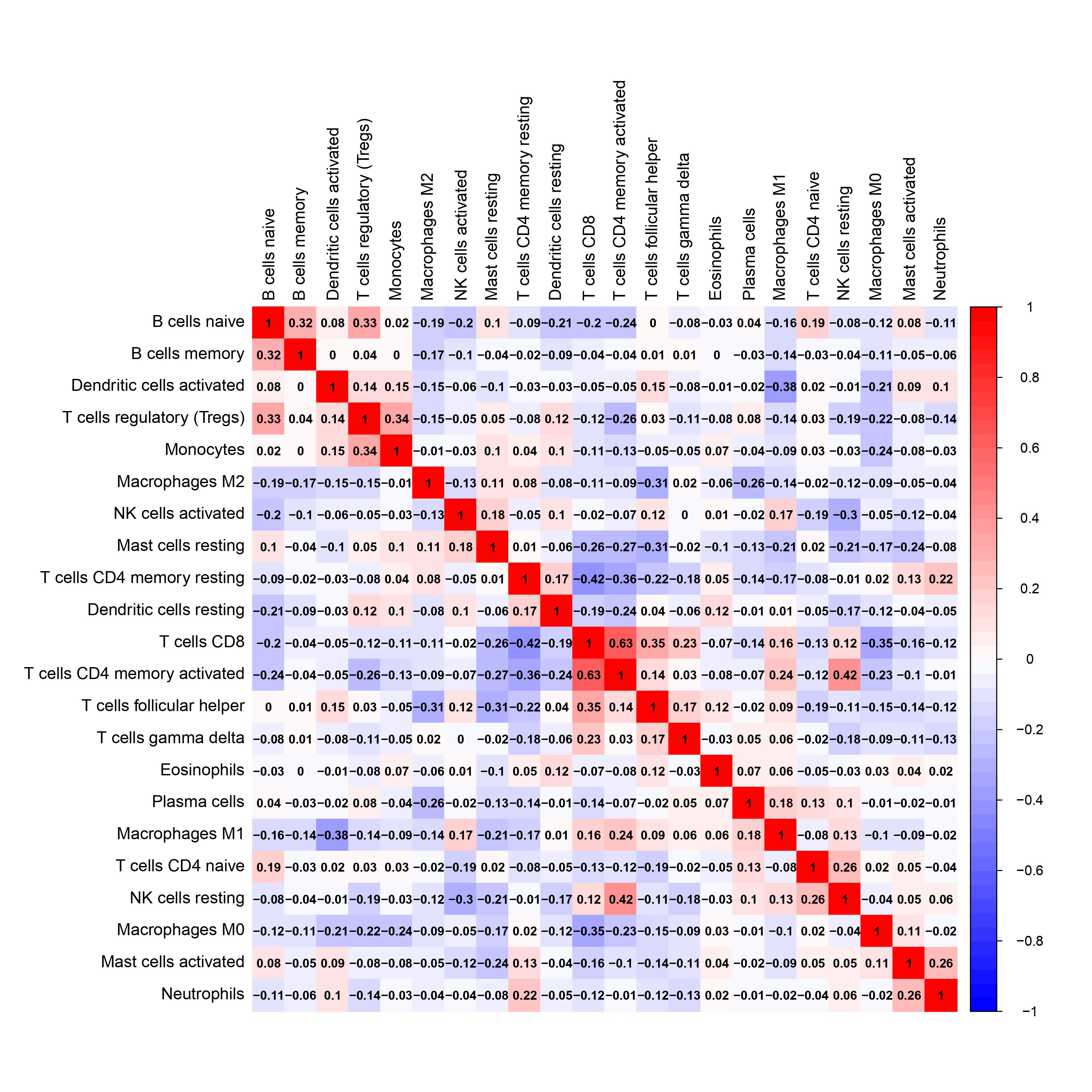
**

**Figure S4.** The correlation between immune cells.

**Figure S5.**


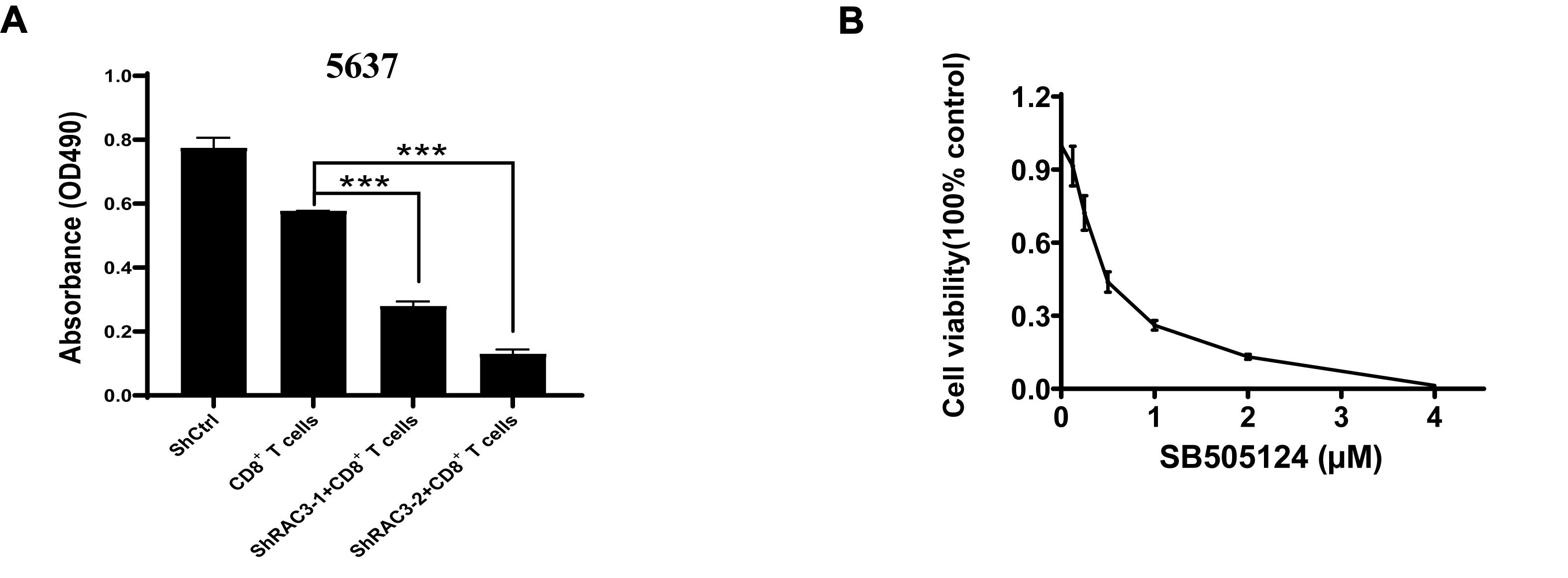


**Figure S5.** Effects of CD8^+^ T cells on BLCA cells in the absence of RAC3 and the effect of SB505124 on BLCA cells. (A) Effect of CD8^+^ T cells on BLCA cell proliferation after knocking out RAC3. (B) Effects of different concentrations of SB505124 on BLCA cell viability.

**Supplementary Table S1.** The clinical characteristics of BLCA patients in the BLCA cohort.

| **Clinical characters** | **Number** |
| --- | --- |
| Gender  Male  Female | 300  106 |
| Age |  |
| Mean (SD) | 68.1 (10.6) |
| Median [MIN, MAX] | 69 [34, 90] |
| TNM stage  I  II  III  IV | 2  129  140  133 |
| pT_stage  T1  T2  T3  T4  TX | 11  190  157  42  6 |
| pN_stage  N0  N1  N2  N3  NX | 236  46  75  7  36 |
| pM_stage  M0  M1  MX | 195  11  197 |

**Supplementary Table S2.** 640 genes were downloaded from the Genecards database and Harmonizome database.

| **Gene Symbol** | **Description** |
| --- | --- |
| BRMS1 | BRMS1 Transcriptional Repressor And Anoikis Regulator |
| PTK2 | Protein Tyrosine Kinase 2 |
| NTRK2 | Neurotrophic Receptor Tyrosine Kinase 2 |
| BCL2L11 | BCL2 Like 11 |
| SRC | SRC Proto-Oncogene, Non-Receptor Tyrosine Kinase |
| CEACAM6 | CEA Cell Adhesion Molecule 6 |
| CAV1 | Caveolin 1 |
| AKT1 | AKT Serine/Threonine Kinase 1 |
| ITGB1 | Integrin Subunit Beta 1 |
| CEACAM5 | CEA Cell Adhesion Molecule 5 |
| EGFR | Epidermal Growth Factor Receptor |
| BCL2 | BCL2 Apoptosis Regulator |
| CASP8 | Caspase 8 |
| SIK1 | Salt Inducible Kinase 1 |
| PTRH2 | Peptidyl-TRNA Hydrolase 2 |
| STAT3 | Signal Transducer And Activator Of Transcription 3 |
| TLE1 | TLE Family Member 1, Transcriptional Corepressor |
| DAPK2 | Death Associated Protein Kinase 2 |
| CTNNB1 | Catenin Beta 1 |
| ZNF304 | Zinc Finger Protein 304 |
| MAPK1 | Mitogen-Activated Protein Kinase 1 |
| BMF | Bcl2 Modifying Factor |
| ITGA5 | Integrin Subunit Alpha 5 |
| TP53 | Tumor Protein P53 |
| MCL1 | MCL1 Apoptosis Regulator, BCL2 Family Member |
| BCL2L1 | BCL2 Like 1 |
| CASP3 | Caspase 3 |
| CDH1 | Cadherin 1 |
| BAD | BCL2 Associated Agonist Of Cell Death |
| PIK3CA | Phosphatidylinositol-4,5-Bisphosphate 3-Kinase Catalytic Subunit Alpha |
| PAK1 | P21 (RAC1) Activated Kinase 1 |
| ITGAV | Integrin Subunit Alpha V |
| FN1 | Fibronectin 1 |
| MAPK3 | Mitogen-Activated Protein Kinase 3 |
| PTGS2 | Prostaglandin-Endoperoxide Synthase 2 |
| BAX | BCL2 Associated X, Apoptosis Regulator |
| BCAR1 | BCAR1 Scaffold Protein, Cas Family Member |
| PTEN | Phosphatase And Tensin Homolog |
| ERBB2 | Erb-B2 Receptor Tyrosine Kinase 2 |
| ANGPTL4 | Angiopoietin Like 4 |
| PDK4 | Pyruvate Dehydrogenase Kinase 4 |
| CYCS | Cytochrome C, Somatic |
| BRAF | B-Raf Proto-Oncogene, Serine/Threonine Kinase |
| YAP1 | Yes1 Associated Transcriptional Regulator |
| ANKRD13C | Ankyrin Repeat Domain 13C |
| ITGA2 | Integrin Subunit Alpha 2 |
| ANXA5 | Annexin A5 |
| BIRC5 | Baculoviral IAP Repeat Containing 5 |
| MTOR | Mechanistic Target Of Rapamycin Kinase |
| TIMP1 | TIMP Metallopeptidase Inhibitor 1 |
| BDNF | Brain Derived Neurotrophic Factor |
| CSPG4 | Chondroitin Sulfate Proteoglycan 4 |
| BSG | Basigin (Ok Blood Group) |
| AKT2 | AKT Serine/Threonine Kinase 2 |
| STK11 | Serine/Threonine Kinase 11 |
| IGF1 | Insulin Like Growth Factor 1 |
| IGF1R | Insulin Like Growth Factor 1 Receptor |
| ITGA6 | Integrin Subunit Alpha 6 |
| ILK | Integrin Linked Kinase |
| CFLAR | CASP8 And FADD Like Apoptosis Regulator |
| RHOA | Ras Homolog Family Member A |
| HIF1A | Hypoxia Inducible Factor 1 Subunit Alpha |
| DAP3 | Death Associated Protein 3 |
| MYBBP1A | MYB Binding Protein 1a |
| TLE5 | TLE Family Member 5, Transcriptional Modulator |
| ITGA3 | Integrin Subunit Alpha 3 |
| PTK2B | Protein Tyrosine Kinase 2 Beta |
| CCND1 | Cyclin D1 |
| CTTN | Cortactin |
| CALR | Calreticulin |
| ATF4 | Activating Transcription Factor 4 |
| CDCP1 | CUB Domain Containing Protein 1 |
| PLAUR | Plasminogen Activator, Urokinase Receptor |
| SKP2 | S-Phase Kinase Associated Protein 2 |
| CHEK2 | Checkpoint Kinase 2 |
| HGF | Hepatocyte Growth Factor |
| E2F1 | E2F Transcription Factor 1 |
| EGF | Epidermal Growth Factor |
| PIK3CG | Phosphatidylinositol-4,5-Bisphosphate 3-Kinase Catalytic Subunit Gamma |
| ITGB4 | Integrin Subunit Beta 4 |
| DAPK1 | Death Associated Protein Kinase 1 |
| MAPK8 | Mitogen-Activated Protein Kinase 8 |
| PIK3R1 | Phosphoinositide-3-Kinase Regulatory Subunit 1 |
| PIK3R3 | Phosphoinositide-3-Kinase Regulatory Subunit 3 |
| MAP2K1 | Mitogen-Activated Protein Kinase Kinase 1 |
| CXCL12 | C-X-C Motif Chemokine Ligand 12 |
| LGALS3 | Galectin 3 |
| FBXW7-AS1 | FBXW7 Antisense RNA 1 |
| BAK1 | BCL2 Antagonist/Killer 1 |
| ABHD4 | Abhydrolase Domain Containing 4, N-Acyl Phospholipase B |
| CD44 | CD44 Molecule (Indian Blood Group) |
| ITGA4 | Integrin Subunit Alpha 4 |
| FADD | Fas Associated Via Death Domain |
| PHLDA2 | Pleckstrin Homology Like Domain Family A Member 2 |
| TGFB1 | Transforming Growth Factor Beta 1 |
| HMCN1 | Hemicentin 1 |
| MMP2 | Matrix Metallopeptidase 2 |
| CEBPB | CCAAT Enhancer Binding Protein Beta |
| CEMIP | Cell Migration Inducing Hyaluronidase 1 |
| CDKN3 | Cyclin Dependent Kinase Inhibitor 3 |
| CBL | Cbl Proto-Oncogene |
| CASP9 | Caspase 9 |
| SFN | Stratifin |
| MTDH | Metadherin |
| PRKCA | Protein Kinase C Alpha |
| TNFRSF10B | TNF Receptor Superfamily Member 10b |
| CXCL8 | C-X-C Motif Chemokine Ligand 8 |
| MIR200C | MicroRNA 200c |
| AR | Androgen Receptor |
| CDKN2A | Cyclin Dependent Kinase Inhibitor 2A |
| CPT1A | Carnitine Palmitoyltransferase 1A |
| PIK3CB | Phosphatidylinositol-4,5-Bisphosphate 3-Kinase Catalytic Subunit Beta |
| CLDN1 | Claudin 1 |
| MIR204 | MicroRNA 204 |
| MIR26A1 | MicroRNA 26a-1 |
| CDKN1A | Cyclin Dependent Kinase Inhibitor 1A |
| CDKN1B | Cyclin Dependent Kinase Inhibitor 1B |
| KLF12 | KLF Transcription Factor 12 |
| NTRK1 | Neurotrophic Receptor Tyrosine Kinase 1 |
| PLAU | Plasminogen Activator, Urokinase |
| MYC | MYC Proto-Oncogene, BHLH Transcription Factor |
| SMAD4 | SMAD Family Member 4 |
| PLK1 | Polo Like Kinase 1 |
| MUC1 | Mucin 1, Cell Surface Associated |
| LGALS1 | Galectin 1 |
| PYCARD | PYD And CARD Domain Containing |
| SESN2 | Sestrin 2 |
| ITGB3 | Integrin Subunit Beta 3 |
| KRAS | KRAS Proto-Oncogene, GTPase |
| THBS1 | Thrombospondin 1 |
| BID | BH3 Interacting Domain Death Agonist |
| HRAS | HRas Proto-Oncogene, GTPase |
| CDK11B | Cyclin Dependent Kinase 11B |
| CDK11A | Cyclin Dependent Kinase 11A |
| XIAP | X-Linked Inhibitor Of Apoptosis |
| PPARG | Peroxisome Proliferator Activated Receptor Gamma |
| IL6 | Interleukin 6 |
| MIR145 | MicroRNA 145 |
| CCR7 | C-C Motif Chemokine Receptor 7 |
| MSLN | Mesothelin |
| RAC1 | Rac Family Small GTPase 1 |
| GRHL2 | Grainyhead Like Transcription Factor 2 |
| BIRC3 | Baculoviral IAP Repeat Containing 3 |
| NOTCH1 | Notch Receptor 1 |
| RHOG | Ras Homolog Family Member G |
| CCAR2 | Cell Cycle And Apoptosis Regulator 2 |
| NQO1 | NAD(P)H Quinone Dehydrogenase 1 |
| MMP13 | Matrix Metallopeptidase 13 |
| FAS | Fas Cell Surface Death Receptor |
| MTA1 | Metastasis Associated 1 |
| MYO5A | Myosin VA |
| EDA2R | Ectodysplasin A2 Receptor |
| CCN6 | Cellular Communication Network Factor 6 |
| MMP9 | Matrix Metallopeptidase 9 |
| ABL1 | ABL Proto-Oncogene 1, Non-Receptor Tyrosine Kinase |
| MAPK11 | Mitogen-Activated Protein Kinase 11 |
| SOD2 | Superoxide Dismutase 2 |
| PTHLH | Parathyroid Hormone Like Hormone |
| PDGFB | Platelet Derived Growth Factor Subunit B |
| GLI2 | GLI Family Zinc Finger 2 |
| EZH2 | Enhancer Of Zeste 2 Polycomb Repressive Complex 2 Subunit |
| RIPK1 | Receptor Interacting Serine/Threonine Kinase 1 |
| CXCR4 | C-X-C Motif Chemokine Receptor 4 |
| HMGA1 | High Mobility Group AT-Hook 1 |
| SIK2 | Salt Inducible Kinase 2 |
| TNFSF10 | TNF Superfamily Member 10 |
| ANGPTL2 | Angiopoietin Like 2 |
| S100A4 | S100 Calcium Binding Protein A4 |
| NTF3 | Neurotrophin 3 |
| ETV4 | ETS Variant Transcription Factor 4 |
| MIR21 | MicroRNA 21 |
| MIR124-1 | MicroRNA 124-1 |
| HTRA1 | HtrA Serine Peptidase 1 |
| LATS1 | Large Tumor Suppressor Kinase 1 |
| CEACAM3 | CEA Cell Adhesion Molecule 3 |
| EIF2AK3 | Eukaryotic Translation Initiation Factor 2 Alpha Kinase 3 |
| LAMC2 | Laminin Subunit Gamma 2 |
| LAMA3 | Laminin Subunit Alpha 3 |
| LAMB3 | Laminin Subunit Beta 3 |
| CDH2 | Cadherin 2 |
| CSNK2A1 | Casein Kinase 2 Alpha 1 |
| EDIL3 | EGF Like Repeats And Discoidin Domains 3 |
| ZEB2 | Zinc Finger E-Box Binding Homeobox 2 |
| TLN1 | Talin 1 |
| EPHA2 | EPH Receptor A2 |
| SIRT3 | Sirtuin 3 |
| OLFM3 | Olfactomedin 3 |
| CLU | Clusterin |
| SPINK1 | Serine Peptidase Inhibitor Kazal Type 1 |
| CPEB2 | Cytoplasmic Polyadenylation Element Binding Protein 2 |
| NAT1 | N-Acetyltransferase 1 |
| TSG101 | Tumor Susceptibility 101 |
| MIR200A | MicroRNA 200a |
| MIR6744 | MicroRNA 6744 |
| SERPINA1 | Serpin Family A Member 1 |
| AKT3 | AKT Serine/Threonine Kinase 3 |
| RELA | RELA Proto-Oncogene, NF-KB Subunit |
| TNFRSF1A | TNF Receptor Superfamily Member 1A |
| FASLG | Fas Ligand |
| AFP | Alpha Fetoprotein |
| ITGA8 | Integrin Subunit Alpha 8 |
| NOX4 | NADPH Oxidase 4 |
| PBK | PDZ Binding Kinase |
| SATB1 | SATB Homeobox 1 |
| CD63 | CD63 Molecule |
| EEF1A1 | Eukaryotic Translation Elongation Factor 1 Alpha 1 |
| LTB4R2 | Leukotriene B4 Receptor 2 |
| MAVS | Mitochondrial Antiviral Signaling Protein |
| HRC | Histidine Rich Calcium Binding Protein |
| CCN2 | Cellular Communication Network Factor 2 |
| RHOB | Ras Homolog Family Member B |
| PPP1R13B | Protein Phosphatase 1 Regulatory Subunit 13B |
| PLG | Plasminogen |
| MET | MET Proto-Oncogene, Receptor Tyrosine Kinase |
| RAF1 | Raf-1 Proto-Oncogene, Serine/Threonine Kinase |
| PARP1 | Poly(ADP-Ribose) Polymerase 1 |
| PRKCQ | Protein Kinase C Theta |
| BRCA2 | BRCA2 DNA Repair Associated |
| RB1 | RB Transcriptional Corepressor 1 |
| SP1 | Sp1 Transcription Factor |
| HAVCR2 | Hepatitis A Virus Cellular Receptor 2 |
| DOCK1 | Dedicator Of Cytokinesis 1 |
| VTN | Vitronectin |
| INHBB | Inhibin Subunit Beta B |
| PDCD4 | Programmed Cell Death 4 |
| PRPF4B | Pre-MRNA Processing Factor 4B |
| RANBP9 | RAN Binding Protein 9 |
| SESN1 | Sestrin 1 |
| SESN3 | Sestrin 3 |
| CD24 | CD24 Molecule |
| ZBTB7A | Zinc Finger And BTB Domain Containing 7A |
| MIR141 | MicroRNA 141 |
| ELANE | Elastase, Neutrophil Expressed |
| KDR | Kinase Insert Domain Receptor |
| MDM2 | MDM2 Proto-Oncogene |
| NFE2L2 | NFE2 Like BZIP Transcription Factor 2 |
| ZEB1 | Zinc Finger E-Box Binding Homeobox 1 |
| KL | Klotho |
| PRKCI | Protein Kinase C Iota |
| CRYAB | Crystallin Alpha B |
| EPHB6 | EPH Receptor B6 |
| FGF2 | Fibroblast Growth Factor 2 |
| HK2 | Hexokinase 2 |
| LTF | Lactotransferrin |
| IQGAP1 | IQ Motif Containing GTPase Activating Protein 1 |
| MGAT5 | Alpha-1,6-Mannosylglycoprotein 6-Beta-N-Acetylglucosaminyltransferase |
| SDCBP | Syndecan Binding Protein |
| ABHD2 | Abhydrolase Domain Containing 2, Acylglycerol Lipase |
| SPIB | Spi-B Transcription Factor |
| TRIM31 | Tripartite Motif Containing 31 |
| MIR1827 | MicroRNA 1827 |
| PDGFRB | Platelet Derived Growth Factor Receptor Beta |
| PLAT | Plasminogen Activator, Tissue Type |
| TLR3 | Toll Like Receptor 3 |
| NRAS | NRAS Proto-Oncogene, GTPase |
| ROCK1 | Rho Associated Coiled-Coil Containing Protein Kinase 1 |
| PAK4 | P21 (RAC1) Activated Kinase 4 |
| VEGFA | Vascular Endothelial Growth Factor A |
| CASP10 | Caspase 10 |
| PIN1 | Peptidylprolyl Cis/Trans Isomerase, NIMA-Interacting 1 |
| IL1RAP | Interleukin 1 Receptor Accessory Protein |
| UBE2C | Ubiquitin Conjugating Enzyme E2 C |
| YWHAZ | Tyrosine 3-Monooxygenase/Tryptophan 5-Monooxygenase Activation Protein Zeta |
| TWIST1 | Twist Family BHLH Transcription Factor 1 |
| BMP6 | Bone Morphogenetic Protein 6 |
| BNIP3L | BCL2 Interacting Protein 3 Like |
| ELK1 | ETS Transcription Factor ELK1 |
| KDM3A | Lysine Demethylase 3A |
| PRDX4 | Peroxiredoxin 4 |
| BNIP3 | BCL2 Interacting Protein 3 |
| LMO3 | LIM Domain Only 3 |
| ZNF32 | Zinc Finger Protein 32 |
| MIR200B | MicroRNA 200b |
| MIR525 | MicroRNA 525 |
| MIR363 | MicroRNA 363 |
| TUBB3 | Tubulin Beta 3 Class III |
| HSP90B1 | Heat Shock Protein 90 Beta Family Member 1 |
| SLC2A1 | Solute Carrier Family 2 Member 1 |
| HMOX1 | Heme Oxygenase 1 |
| PTPN11 | Protein Tyrosine Phosphatase Non-Receptor Type 11 |
| PRKACA | Protein Kinase CAMP-Activated Catalytic Subunit Alpha |
| PAK3 | P21 (RAC1) Activated Kinase 3 |
| CD36 | CD36 Molecule |
| PIK3R2 | Phosphoinositide-3-Kinase Regulatory Subunit 2 |
| PPP2CA | Protein Phosphatase 2 Catalytic Subunit Alpha |
| CASP6 | Caspase 6 |
| CDH3 | Cadherin 3 |
| EEF2K | Eukaryotic Elongation Factor 2 Kinase |
| LRP1 | LDL Receptor Related Protein 1 |
| PAK2 | P21 (RAC1) Activated Kinase 2 |
| PTK6 | Protein Tyrosine Kinase 6 |
| LPAR1 | Lysophosphatidic Acid Receptor 1 |
| TCF7L2 | Transcription Factor 7 Like 2 |
| CEACAM1 | CEA Cell Adhesion Molecule 1 |
| GDF2 | Growth Differentiation Factor 2 |
| GLO1 | Glyoxalase I |
| IL17A | Interleukin 17A |
| RBL2 | RB Transcriptional Corepressor Like 2 |
| SIRPA | Signal Regulatory Protein Alpha |
| TRAF2 | TNF Receptor Associated Factor 2 |
| ADCY10 | Adenylate Cyclase 10 |
| VPS37A | VPS37A Subunit Of ESCRT-I |
| TNFRSF12A | TNF Receptor Superfamily Member 12A |
| APOBEC3G | Apolipoprotein B MRNA Editing Enzyme Catalytic Subunit 3G |
| BAG1 | BAG Cochaperone 1 |
| COL13A1 | Collagen Type XIII Alpha 1 Chain |
| MNX1 | Motor Neuron And Pancreas Homeobox 1 |
| RAD9A | RAD9 Checkpoint Clamp Component A |
| IFI27 | Interferon Alpha Inducible Protein 27 |
| MEGF11 | Multiple EGF Like Domains 11 |
| ITPRIP | Inositol 1,4,5-Trisphosphate Receptor Interacting Protein |
| BCL2L15 | BCL2 Like 15 |
| SNAI2 | Snail Family Transcriptional Repressor 2 |
| PTPN1 | Protein Tyrosine Phosphatase Non-Receptor Type 1 |
| NOTCH3 | Notch Receptor 3 |
| GLUD1 | Glutamate Dehydrogenase 1 |
| SIRT1 | Sirtuin 1 |
| FASN | Fatty Acid Synthase |
| MYH9 | Myosin Heavy Chain 9 |
| RPS6KB1 | Ribosomal Protein S6 Kinase B1 |
| TPM1 | Tropomyosin 1 |
| PPP2R1A | Protein Phosphatase 2 Scaffold Subunit Aalpha |
| COL4A2 | Collagen Type IV Alpha 2 Chain |
| CTNND1 | Catenin Delta 1 |
| CD151 | CD151 Molecule (Raph Blood Group) |
| MMP11 | Matrix Metallopeptidase 11 |
| ARHGEF7 | Rho Guanine Nucleotide Exchange Factor 7 |
| PPP2R2A | Protein Phosphatase 2 Regulatory Subunit Balpha |
| SEMA7A | Semaphorin 7A (John Milton Hagen Blood Group) |
| PPP2R5A | Protein Phosphatase 2 Regulatory Subunit B'Alpha |
| BST2 | Bone Marrow Stromal Cell Antigen 2 |
| CCN1 | Cellular Communication Network Factor 1 |
| PPP2R2D | Protein Phosphatase 2 Regulatory Subunit Bdelta |
| CCDC178 | Coiled-Coil Domain Containing 178 |
| MIR10A | MicroRNA 10a |
| MIR30B | MicroRNA 30b |
| MIR30C1 | MicroRNA 30c-1 |
| SHC1 | SHC Adaptor Protein 1 |
| BUB1 | BUB1 Mitotic Checkpoint Serine/Threonine Kinase |
| CDC25C | Cell Division Cycle 25C |
| CDK1 | Cyclin Dependent Kinase 1 |
| ITGB5 | Integrin Subunit Beta 5 |
| SETD2 | SET Domain Containing 2, Histone Lysine Methyltransferase |
| BUB3 | BUB3 Mitotic Checkpoint Protein |
| FER | FER Tyrosine Kinase |
| TP73 | Tumor Protein P73 |
| SLCO1B3 | Solute Carrier Organic Anion Transporter Family Member 1B3 |
| TDGF1 | Teratocarcinoma-Derived Growth Factor 1 |
| DLG1 | Discs Large MAGUK Scaffold Protein 1 |
| EDAR | Ectodysplasin A Receptor |
| MAD2L1 | Mitotic Arrest Deficient 2 Like 1 |
| BCL2L2 | BCL2 Like 2 |
| PDCD6IP | Programmed Cell Death 6 Interacting Protein |
| SH3GLB1 | SH3 Domain Containing GRB2 Like, Endophilin B1 |
| SCRIB | Scribble Planar Cell Polarity Protein |
| DYNLL2 | Dynein Light Chain LC8-Type 2 |
| TSC2 | TSC Complex Subunit 2 |
| BAG4 | BAG Cochaperone 4 |
| MAP3K7 | Mitogen-Activated Protein Kinase Kinase Kinase 7 |
| F10 | Coagulation Factor X |
| F3 | Coagulation Factor III, Tissue Factor |
| ADAMTSL1 | ADAMTS Like 1 |
| SERPINB1 | Serpin Family B Member 1 |
| MIR181A1 | MicroRNA 181a-1 |
| MAP3K1 | Mitogen-Activated Protein Kinase Kinase Kinase 1 |
| CTBP1 | C-Terminal Binding Protein 1 |
| CEACAM4 | CEA Cell Adhesion Molecule 4 |
| PXN | Paxillin |
| MALAT1 | Metastasis Associated Lung Adenocarcinoma Transcript 1 |
| GSTP1 | Glutathione S-Transferase Pi 1 |
| PRDX1 | Peroxiredoxin 1 |
| IKBKG | Inhibitor Of Nuclear Factor Kappa B Kinase Regulatory Subunit Gamma |
| TFDP1 | Transcription Factor Dp-1 |
| CRYBA1 | Crystallin Beta A1 |
| SERPINE1 | Serpin Family E Member 1 |
| FOXO3 | Forkhead Box O3 |
| ACTG1 | Actin Gamma 1 |
| ARHGDIA | Rho GDP Dissociation Inhibitor Alpha |
| EZR | Ezrin |
| SLC39A6 | Solute Carrier Family 39 Member 6 |
| BIN1 | Bridging Integrator 1 |
| TIAM1 | TIAM Rac1 Associated GEF 1 |
| PDPK1 | 3-Phosphoinositide Dependent Protein Kinase 1 |
| SMAD7 | SMAD Family Member 7 |
| NTRK3 | Neurotrophic Receptor Tyrosine Kinase 3 |
| RHOC | Ras Homolog Family Member C |
| CASP2 | Caspase 2 |
| TNC | Tenascin C |
| IRF6 | Interferon Regulatory Factor 6 |
| HOTAIR | HOX Transcript Antisense RNA |
| GNE | Glucosamine (UDP-N-Acetyl)-2-Epimerase/N-Acetylmannosamine Kinase |
| XAF1 | XIAP Associated Factor 1 |
| SFRP1 | Secreted Frizzled Related Protein 1 |
| MAP2K2 | Mitogen-Activated Protein Kinase Kinase 2 |
| CSK | C-Terminal Src Kinase |
| PIK3C2B | Phosphatidylinositol-4-Phosphate 3-Kinase Catalytic Subunit Type 2 Beta |
| TAGLN | Transgelin |
| ENDOG | Endonuclease G |
| FOXC2 | Forkhead Box C2 |
| RACK1 | Receptor For Activated C Kinase 1 |
| ARHGDIB | Rho GDP Dissociation Inhibitor Beta |
| FBLIM1 | Filamin Binding LIM Protein 1 |
| CCDC80 | Coiled-Coil Domain Containing 80 |
| PRKD1 | Protein Kinase D1 |
| LDHA | Lactate Dehydrogenase A |
| ANXA2 | Annexin A2 |
| SPP1 | Secreted Phosphoprotein 1 |
| SMARCE1 | SWI/SNF Related, Matrix Associated, Actin Dependent Regulator Of Chromatin, Subfamily E, Member 1 |
| QSOX1 | Quiescin Sulfhydryl Oxidase 1 |
| RBFOX2 | RNA Binding Fox-1 Homolog 2 |
| RPS6KA3 | Ribosomal Protein S6 Kinase A3 |
| CDC42 | Cell Division Cycle 42 |
| MAOA | Monoamine Oxidase A |
| PIP5K1C | Phosphatidylinositol-4-Phosphate 5-Kinase Type 1 Gamma |
| ATF2 | Activating Transcription Factor 2 |
| JUP | Junction Plakoglobin |
| NDRG1 | N-Myc Downstream Regulated 1 |
| NKX2-1 | NK2 Homeobox 1 |
| OCLN | Occludin |
| CRABP2 | Cellular Retinoic Acid Binding Protein 2 |
| ID2 | Inhibitor Of DNA Binding 2 |
| CEACAM8 | CEA Cell Adhesion Molecule 8 |
| PITPNC1 | Phosphatidylinositol Transfer Protein Cytoplasmic 1 |
| AFAP1L1 | Actin Filament Associated Protein 1 Like 1 |
| INSR | Insulin Receptor |
| HSPB1 | Heat Shock Protein Family B (Small) Member 1 |
| NGF | Nerve Growth Factor |
| PCNA | Proliferating Cell Nuclear Antigen |
| GSK3B | Glycogen Synthase Kinase 3 Beta |
| TP63 | Tumor Protein P63 |
| KRT14 | Keratin 14 |
| SPHK1 | Sphingosine Kinase 1 |
| CTNNA1 | Catenin Alpha 1 |
| EHMT2 | Euchromatic Histone Lysine Methyltransferase 2 |
| OGT | O-Linked N-Acetylglucosamine (GlcNAc) Transferase |
| RAC3 | Rac Family Small GTPase 3 |
| SIRT6 | Sirtuin 6 |
| ACP1 | Acid Phosphatase 1 |
| FOXA1 | Forkhead Box A1 |
| STK38 | Serine/Threonine Kinase 38 |
| RHOQ | Ras Homolog Family Member Q |
| ONECUT1 | One Cut Homeobox 1 |
| S100A7 | S100 Calcium Binding Protein A7 |
| SRSF3 | Serine And Arginine Rich Splicing Factor 3 |
| MUC4 | Mucin 4, Cell Surface Associated |
| GKN1 | Gastrokine 1 |
| MIR107 | MicroRNA 107 |
| MIR630 | MicroRNA 630 |
| DNMT1 | DNA Methyltransferase 1 |
| LCK | LCK Proto-Oncogene, Src Family Tyrosine Kinase |
| MERTK | MER Proto-Oncogene, Tyrosine Kinase |
| UCHL1 | Ubiquitin C-Terminal Hydrolase L1 |
| CDK2 | Cyclin Dependent Kinase 2 |
| MMP3 | Matrix Metallopeptidase 3 |
| ACTB | Actin Beta |
| BRCA1 | BRCA1 DNA Repair Associated |
| SLC2A2 | Solute Carrier Family 2 Member 2 |
| NOS2 | Nitric Oxide Synthase 2 |
| USP9X | Ubiquitin Specific Peptidase 9 X-Linked |
| ROR1 | Receptor Tyrosine Kinase Like Orphan Receptor 1 |
| FYN | FYN Proto-Oncogene, Src Family Tyrosine Kinase |
| HSPA1A | Heat Shock Protein Family A (Hsp70) Member 1A |
| HTRA2 | HtrA Serine Peptidase 2 |
| SNAI1 | Snail Family Transcriptional Repressor 1 |
| C5AR1 | Complement C5a Receptor 1 |
| LATS2 | Large Tumor Suppressor Kinase 2 |
| PRDM1 | PR/SET Domain 1 |
| SKI | SKI Proto-Oncogene |
| TPP2 | Tripeptidyl Peptidase 2 |
| XRCC5 | X-Ray Repair Cross Complementing 5 |
| CLDN18 | Claudin 18 |
| SPTA1 | Spectrin Alpha, Erythrocytic 1 |
| THY1 | Thy-1 Cell Surface Antigen |
| TJP1 | Tight Junction Protein 1 |
| CDX2 | Caudal Type Homeobox 2 |
| CENPF | Centromere Protein F |
| DOK2 | Docking Protein 2 |
| S100A11 | S100 Calcium Binding Protein A11 |
| SERPINB5 | Serpin Family B Member 5 |
| CLIC4 | Chloride Intracellular Channel 4 |
| IKZF3 | IKAROS Family Zinc Finger 3 |
| SNCG | Synuclein Gamma |
| USP11 | Ubiquitin Specific Peptidase 11 |
| ELAVL1 | ELAV Like RNA Binding Protein 1 |
| HOXA10 | Homeobox A10 |
| LGALS8 | Galectin 8 |
| SRPX2 | Sushi Repeat Containing Protein X-Linked 2 |
| SLPI | Secretory Leukocyte Peptidase Inhibitor |
| HTRA3 | HtrA Serine Peptidase 3 |
| EFHD2 | EF-Hand Domain Family Member D2 |
| IRX1 | Iroquois Homeobox 1 |
| CXCL14 | C-X-C Motif Chemokine Ligand 14 |
| KIF18A | Kinesin Family Member 18A |
| ZG16B | Zymogen Granule Protein 16B |
| SBSN | Suprabasin |
| MIR223 | MicroRNA 223 |
| MIR503 | MicroRNA 503 |
| MIR99A | MicroRNA 99a |
| MIR451A | MicroRNA 451a |
| MIR7-1 | MicroRNA 7-1 |
| SNORA80E | Small Nucleolar RNA, H/ACA Box 80E |
| BMF | Bcl2 modifying factor |
| DAPK2 | death-associated protein kinase 2 |
| E2F1 | E2F transcription factor 1 |
| STK11 | serine/threonine kinase 11 |
| TFDP1 | transcription factor Dp-1 |
| ABHD4 | abhydrolase domain containing 4 |
| AFAP1L1 | actin filament associated protein 1-like 1 |
| AKT1 | v-akt murine thymoma viral oncogene homolog 1 |
| AKT2 | v-akt murine thymoma viral oncogene homolog 2 |
| ANGPTL4 | angiopoietin-like 4 |
| BAX | BCL2-associated X protein |
| BCAR1 | breast cancer anti-estrogen resistance 1 |
| BCL2 | B-cell CLL/lymphoma 2 |
| BCL2L11 | BCL2-like 11 (apoptosis facilitator) |
| BIRC3 | baculoviral IAP repeat containing 3 |
| BMF | Bcl2 modifying factor |
| BRAF | B-Raf proto-oncogene, serine/threonine kinase |
| BRCA2 | breast cancer 2, early onset |
| BSG | basigin (Ok blood group) |
| CALR | calreticulin |
| CASP2 | caspase 2, apoptosis-related cysteine peptidase |
| CASP3 | caspase 3, apoptosis-related cysteine peptidase |
| CASP8 | caspase 8, apoptosis-related cysteine peptidase |
| CAV1 | caveolin 1, caveolae protein, 22kDa |
| CCAR2 | cell cycle and apoptosis regulator 2 |
| CD63 | CD63 molecule |
| CDCP1 | CUB domain containing protein 1 |
| CDH1 | cadherin 1, type 1, E-cadherin (epithelial) |
| CDH2 | cadherin 2, type 1, N-cadherin (neuronal) |
| CDKN2A | cyclin-dependent kinase inhibitor 2A |
| CEACAM6 | carcinoembryonic antigen-related cell adhesion molecule 6 (non-specific cross reacting antigen) |
| CEBPB | CCAAT/enhancer binding protein (C/EBP), beta |
| CHUK | conserved helix-loop-helix ubiquitous kinase |
| CLDN1 | claudin 1 |
| CLU | clusterin |
| CMA1 | chymase 1, mast cell |
| COPS5 | COP9 signalosome subunit 5 |
| CSNK2A1 | casein kinase 2, alpha 1 polypeptide |
| CSPG4 | chondroitin sulfate proteoglycan 4 |
| CTNND1 | catenin (cadherin-associated protein), delta 1 |
| CTTN | cortactin |
| CXCL12 | chemokine (C-X-C motif) ligand 12 |
| DAP3 | death associated protein 3 |
| DAPK1 | death-associated protein kinase 1 |
| DLG1 | discs, large homolog 1 (Drosophila) |
| EDA2R | ectodysplasin A2 receptor |
| EEF1A1 | eukaryotic translation elongation factor 1 alpha 1 |
| EEF2K | eukaryotic elongation factor 2 kinase |
| EGFR | epidermal growth factor receptor |
| EIF2AK3 | eukaryotic translation initiation factor 2-alpha kinase 3 |
| ERBB4 | erb-b2 receptor tyrosine kinase 4 |
| FER | fer (fps/fes related) tyrosine kinase |
| FGF2 | fibroblast growth factor 2 (basic) |
| FN1 | fibronectin 1 |
| HGF | hepatocyte growth factor (hepapoietin A; scatter factor) |
| HK2 | hexokinase 2 |
| HMCN1 | hemicentin 1 |
| HMGA1 | high mobility group AT-hook 1 |
| HOXA10 | homeobox A10 |
| HTRA1 | HtrA serine peptidase 1 |
| IGF1R | insulin-like growth factor 1 receptor |
| IKZF3 | IKAROS family zinc finger 3 (Aiolos) |
| ITGA2 | integrin, alpha 2 (CD49B, alpha 2 subunit of VLA-2 receptor) |
| ITGA3 | integrin, alpha 3 (antigen CD49C, alpha 3 subunit of VLA-3 receptor) |
| ITGA4 | integrin, alpha 4 (antigen CD49D, alpha 4 subunit of VLA-4 receptor) |
| ITGA5 | integrin, alpha 5 (fibronectin receptor, alpha polypeptide) |
| ITGA6 | integrin, alpha 6 |
| ITGA8 | integrin, alpha 8 |
| ITGAV | integrin, alpha V |
| ITGB1 | integrin, beta 1 (fibronectin receptor, beta polypeptide, antigen CD29 includes MDF2, MSK12) |
| KDR | kinase insert domain receptor |
| KL | klotho |
| KRAS | Kirsten rat sarcoma viral oncogene homolog |
| LGALS1 | lectin, galactoside-binding, soluble, 1 |
| LRP1 | low density lipoprotein receptor-related protein 1 |
| LTB4R2 | leukotriene B4 receptor 2 |
| MAPK1 | mitogen-activated protein kinase 1 |
| MAPK3 | mitogen-activated protein kinase 3 |
| MAVS | mitochondrial antiviral signaling protein |
| MCL1 | myeloid cell leukemia 1 |
| MDM2 | MDM2 proto-oncogene, E3 ubiquitin protein ligase |
| MET | MET proto-oncogene, receptor tyrosine kinase |
| MGAT5 | mannosyl (alpha-1,6-)-glycoprotein beta-1,6-N-acetyl-glucosaminyltransferase |
| MIR200C | microRNA 200c |
| MMP11 | matrix metallopeptidase 11 |
| MMP13 | matrix metallopeptidase 13 |
| MMP2 | matrix metallopeptidase 2 |
| MTA1 | metastasis associated 1 |
| MTOR | mechanistic target of rapamycin (serine/threonine kinase) |
| MYBBP1A | MYB binding protein (P160) 1a |
| NRP1 | neuropilin 1 |
| NTF3 | neurotrophin 3 |
| NTRK2 | neurotrophic tyrosine kinase, receptor, type 2 |
| OLFM3 | olfactomedin 3 |
| PAK1 | p21 protein (Cdc42/Rac)-activated kinase 1 |
| PAK4 | p21 protein (Cdc42/Rac)-activated kinase 4 |
| PECAM1 | platelet/endothelial cell adhesion molecule 1 |
| PIK3CA | phosphatidylinositol-4,5-bisphosphate 3-kinase, catalytic subunit alpha |
| PIK3CG | phosphatidylinositol-4,5-bisphosphate 3-kinase, catalytic subunit gamma |
| PLK1 | polo-like kinase 1 |
| PRKCA | protein kinase C, alpha |
| PRKD1 | protein kinase D1 |
| PTEN | phosphatase and tensin homolog |
| PTHLH | parathyroid hormone-like hormone |
| PTK2 | protein tyrosine kinase 2 |
| PTK2B | protein tyrosine kinase 2 beta |
| PTK6 | protein tyrosine kinase 6 |
| PTPN11 | protein tyrosine phosphatase, non-receptor type 11 |
| PTRH2 | peptidyl-tRNA hydrolase 2 |
| RAD9A | RAD9 homolog A (S. pombe) |
| RHOA | ras homolog family member A |
| RHOC | ras homolog family member C |
| RIPK1 | receptor (TNFRSF)-interacting serine-threonine kinase 1 |
| ROCK1 | Rho-associated, coiled-coil containing protein kinase 1 |
| S100A4 | S100 calcium binding protein A4 |
| SCRIB | scribbled planar cell polarity protein |
| SH3GLB1 | SH3-domain GRB2-like endophilin B1 |
| SIK1 | salt-inducible kinase 1 |
| SIRPA | signal-regulatory protein alpha |
| SIRT3 | sirtuin 3 |
| SKP2 | S-phase kinase-associated protein 2, E3 ubiquitin protein ligase |
| SLCO1B3 | solute carrier organic anion transporter family, member 1B3 |
| SMAD4 | SMAD family member 4 |
| SNAI2 | snail family zinc finger 2 |
| SRC | SRC proto-oncogene, non-receptor tyrosine kinase |
| STAT3 | signal transducer and activator of transcription 3 (acute-phase response factor) |
| STK11 | serine/threonine kinase 11 |
| TAGLN | transgelin |
| TGFB1 | transforming growth factor, beta 1 |
| THBS1 | thrombospondin 1 |
| TIMP1 | TIMP metallopeptidase inhibitor 1 |
| TP53 | tumor protein p53 |
| TPM1 | tropomyosin 1 (alpha) |
| UCHL1 | ubiquitin carboxyl-terminal esterase L1 (ubiquitin thiolesterase) |
| USP9X | ubiquitin specific peptidase 9, X-linked |
| WISP3 | WNT1 inducible signaling pathway protein 3 |
| WNT2 | wingless-type MMTV integration site family member 2 |
| XIAP | X-linked inhibitor of apoptosis, E3 ubiquitin protein ligase |
| YWHAZ | tyrosine 3-monooxygenase/tryptophan 5-monooxygenase activation protein, zeta |

**Supplementary Table S3.** Primary antibodies used in this study.

| **Name** | **Supplier** | **Cat no.** |
| --- | --- | --- |
| RAC3 | Abcam | ab129062 |
| GAPDH | Cell Signaling Technology | 2118 |
| Ki67 | Cell Signaling Technology | 9449 |
| PCNA | Cell Signaling Technology | 13110 |
| E-Cadherin | Cell Signaling Technology | 3195 |

**Supplementary Table S4.** The targeting oligos of shRAC3.

| **shRNAs** | **Sequences** |
| --- | --- |
| Sh RAC3-1 | TGACGTCTTTCTGATCTGCTT |
| Sh RAC3-2 | CCGGGAGATTGGCTCTGTGAA |

**Supplementary Table S5.** Nine ARGs were selected by multivariate Cox results.

| **ARGs** | **coef** |
| --- | --- |
| CSPG4 | 0.190927502473781 |
| GLI2 | 0.281101473536596 |
| HMGA1 | 0.356350705812734 |
| NTF3 | -0.429163094370077 |
| CRYAB | 0.155793970084206 |
| RAD9A | -0.558367444114141 |
| FASN | 0.380084107641552 |
| SPP1 | -0.100034977690286 |
| RAC3 | 0.180416238704028 |

ARGs, anoikis-related genes; Coef, coefficient.

**Supplementary Table S6.** Detailed IC50-values for the 60 drugs.
